# Understanding trends in *Zostera* research, stressors, and response variables: a global systematic review of the seagrass genus

**DOI:** 10.7717/peerj.19209

**Published:** 2025-04-17

**Authors:** Hannah R. Lyford, Emma Guerrini Romano, Shayna A. Sura, Sarah Joy Bittick

**Affiliations:** 1 Department of Ecology, Evolution, & Marine Biology, University of California, Santa Barbara, Santa Barbara, CA, United States of America; 2 Biology Department, Loyola Marymount University, Los Angeles, CA, United States of America; 3 Coastal Research Institute, Loyola Marymount University, Los Angeles, CA, United States of America; 4 Department of Ecology and Evolutionary Biology, University of California, Los Angeles, Los Angeles, CA, United States of America

**Keywords:** Seagrass, *Zostera*, Systematic review, Stressors, Response variables, Global, Coastal ecosystem

## Abstract

**Background:**

Seagrass meadows are ecologically significant habitats that are globally threatened. Thus, there is increased interest in conservation of seagrasses as they face widespread decline. Biotic and abiotic factors that influence seagrass can be classified as stressors, such as rising temperature and eutrophication. Our study met an imminent need to consolidate data from previous studies to discern knowledge gaps and identify trends in studies, stressors, species, and geographic origination of research for the genus *Zostera*. For our systematic review, the objectives were to (A) qualitatively assess and summarize the current state of literature focused on seagrass species within the *Zostera* genus and their stressors; (B) utilize data extracted from full-text articles to identify trends and knowledge gaps for the study of stressors, response variable measurements, species, geography, and study designs; and (C) map the distribution, type, and number of these studies globally.

**Methodology:**

We included articles that focused on stressors associated with *Zostera* seagrass species, and excluded studies of other seagrasses and non-stressor related articles. We conducted a Web of Science search of all databases, concluding in January of 2021, followed by a standardized review and data extraction protocol using Colandr (colandrapp.com) as our article screening tool. All 15 review participants were trained on the same set of practice articles and decision trees to minimize variation between individuals. After full text extraction, we analyzed our data by frequency and association between species, stressors, and geographic locations studied.

**Results:**

We screened 7,331 titles and abstracts and extracted data from 1,098 full-text articles. We found nutrients, temperature, and light were the most studied stressors. The United States of America produced the most articles in our review, followed by Australia. *Zostera marina* was most frequently studied, and our review found no stressor studies for five species in the genus. Studies most frequently measured response variables across multiple levels of ecological organization, including the individual plant, biotic community, and environmental conditions. As a part of our review, we made all extracted data publicly available as an interactive map.

**Conclusion:**

Undertaking a review of global studies allowed us to assess more seagrass articles for a single genus than any prior systematic review, summarizing a breadth of stressor studies related to the* Zostera* genus. A team effort and standardized training minimized bias during screening and data extraction. Evidence limitations may exist due to the single database used in our search protocol, as well as species, geographic, and stressor biases in included studies. Our review creates a centralized knowledge base that serves as a foundational information source for *Zostera* research, while highlighting existing knowledge gaps in the literature.

## Introduction

Seagrasses are marine angiosperms that occur off the coasts of every continent besides Antarctica, giving them an almost cosmopolitan distribution (Short et al., 2007). With 13 genera and 72 species known globally, these marine macrophytes are a crucial component of marine ecosystems, particularly along coasts (Short et al., 2011; Nordlund et al., 2016). Seagrass habitats have gained traction as a target for conservation and restoration in recent years due to the increasing losses meadows are experiencing worldwide. It is estimated that 5,602 km^2^ of globally surveyed meadow area has been lost since 1880, which is approximately 19.1% of the meadow area across all bioregions (Dunic et al., 2021). Motivations for seagrass conservation initially focused on economic losses that would ensue from seagrass depletion, particularly in the fisheries industry (Hook et al., 1988). However, it is now recognized the impacts of seagrass loss extend far wider than meadow disappearance alone, threatening to de-stabilize food webs and diminish ecosystem resilience to future disturbances (Kendrick et al., 2019). Seagrasses play valuable roles as marine carbon sinks, erosion controls, provision of coastal protection and water purification, along with supporting commercially relevant fisheries (Tuya, Haroun & Espino, 2014; Orth et al., 2020). Although seagrass meadows have high value and interest has increased, they are an understudied coastal ecosystem (Unsworth, Nordlund & Cullen-Unsworth, 2019). For example, phylogenetic classification and data on seagrass distributions continue to fluctuate as few studies contain thorough data on genetic diversity, range, and descriptions of each genus and species (McKenzie et al., 2020). Also, compared to other ecosystems, seagrasses have low public awareness that slows movement to conserve existing meadows (Ruiz-Frau et al., 2017). Given the increased losses, tremendous environmental and economic value, as well as incomplete distribution data for seagrasses, it is vital that we increase efforts to better understand the stressors contributing to their losses and collate existing datasets to direct future research efforts.

Seagrass losses are largely attributed to stressors, such as water pollution, poor water quality, coastal development, ocean warming and acidification, increased wave energy, and decreased light availability (Griffiths, Connolly & Brown, 2020; Tang & Hadibarata, 2022). These losses often originate from anthropogenic sources; however, stressors can occur naturally as well (Turschwell et al., 2021). Successful conservation and management of seagrasses requires knowledge of which stressors are the most prevalent or will most severely impact seagrasses. Yet, management strategies that historically addressed a single stressor were often not effective in mitigating seagrass losses (Griffiths, Connolly & Brown, 2020). Thus, ongoing and future seagrass management plans will also need to consider the occurrence of multiple simultaneous stressors, which can impact seagrasses differently compared to stressors that occur individually (Unsworth et al., 2015; Lefcheck et al., 2017). To understand how various stressors impact seagrasses, it is important to concentrate existing research on which stressors have been investigated for specific seagrass species, and where these studies have occurred. Summarizing existing data on seagrass stressors will help integrate fundamental research with management policies and legislative actions (Scarano, 2017), improving the success of intervention.

Not only is synthesis of seagrass stressor research needed, but other critical study components will help to provide more holistic knowledge of trends in the study of seagrass ecosystems. These components include study design—for more information on methods used to study species and stressors; distribution of studies—to disentangle any relationship between geography and species or stressors; response variables—to inform how studies are measuring outcomes or indicators; and taxonomy—to gauge frequency of study and consensus in naming for species within a genus. The field of conservation biology has placed an emphasis on considering experimental designs in evidence-based conservation and use of response variables as potential indicators, therefore, we sought to record this data to enhance the utility of our review for researchers, as well as conservation and management practitioners (Cooke et al., 2017; Unsworth et al., 2018). Furthermore, an understanding of geography and taxonomy is limited in published seagrass research, and there have been calls to improve this body of knowledge (Brown et al., 2014; York et al., 2017). It is important to understand the distributions of seagrass studies, and this may also assist in generating an awareness of where publication and research biases lie (Nakagawa et al., 2023). Stressors are one aspect of studies that can be of great interest, especially given recent losses. However, by documenting a variety of study elements, multifaceted study data can provide a more comprehensive picture of how seagrasses have been studied, linking study designs, geography, and response variables with seagrass stressors.

Successful management and holistic research will be vital to the persistence of these valuable seagrass ecosystems. However, past failed actions highlight why conservation actions might consider utilizing or co-creating scientific research in the management decision-making process (Kadykalo et al., 2021). There is an established gap between researchers and practitioners in conservation science due to multiple factors, such as article inaccessibility to practitioners, tradition of using anecdotal evidence in management, low communication between the fields, and barriers brought on by skepticism of information that challenges existing world views (Sunderland et al., 2009; Sutherland et al., 2019). Existence of these gaps and barriers pose problems for plans seeking to employ evidence-based management (Walsh et al., 2019). Seagrass meadows are one such ecosystem that experience failed management actions and lack a centralized framework for addressing losses. A review of 20 case studies in seagrass management found countries including Singapore, Thailand, and Mozambique had poor knowledge of stressors and only certain areas in three countries (Australia, USA, Canada) had policies for managing cumulative impacts of multiple stressors (Griffiths, Connolly & Brown, 2020). Ecosystem management and conservation actions can be a highly complex and variable undertaking, and lack of science translation is not always the underlying reason, or only reason for failure (Maas, Toomey & Loyola, 2019). However, evidence-based management is one approach that can be utilized by practitioners which may increase the success of their projects. A synthesis of seagrass research, including an open access map featuring geographical locations with stressors studied by country and species of interest, will assist in creating better open-access tools for management implementations that adequately address stressors for the location and species present.

The wide range and diversity of species within the *Zostera* genus made it an ideal choice for our study, particularly with our focus on stressors and how their patterns of study may vary on a global scale. *Zostera,* one of the 13 genera of seagrass, is the second most widely distributed (after *Ruppia*) and dominates much of the range that it occupies (Short et al., 2007; Govaerts, 2022). Its unique attributes include high phenotypic plasticity and inter-specific variation, a history of wasting disease, high light requirements, and an ability to grow in a range of climates (Dennison et al., 1993; Short et al., 2007; Bertelli & Unsworth, 2014; Pazzaglia et al., 2021). *Zostera* contains 9 to 18 species based on different sources and is second to *Halophila* for the most species-rich genus (Larkum et al., 2006; Govaerts, 2022). Previous articles, such as Short et al. (2007), assert there are nine species of *Zostera* in existence; however, recent studies suggest up to 18 species may exist, given advances in mapping, data centralization, and molecular methods (Govaerts, 2022; Sullivan & Short, 2023). Based on existing literature sources, 18 species are described for *Zostera*, varying in their distribution, IUCN status, and population trend (Table 1). Studies of different species in the genus have indicated sensitivities to specific stressors, such as salinity (*Z. japonica*), light and temperature (*Z. marina*), and sedimentation (*Z. muelleri*), demonstrating the variable inter-specific nature of the genus (Moore, Shields & Parrish, 2014; Siciliano, Schiel & Thomsen, 2019; Hou et al., 2020). Given the unique characteristics of *Zostera* seagrasses, this genus is a great candidate for synthesizing existing research to broaden our understanding of patterns for species, stressors, and response variables within a single genus on a global scale.

**Table 1:** *Zostera* species descriptions. Distribution, hemisphere, IUCN status, population trend, and descriptive citation for all species within the *Zostera* genus.

**Zostera species**	**Hemisphere**	**Distribution**	**IUCN status**	**Population trend**	**Citation**
*Z. angustifolia*	Northern	northern Europe coastlines, eastern Russia	Not Listed	N/A	Govaerts (2022)
*Z. asiatica*	Northern	eastern Asia	Near Threatened	Decreasing	Govaerts (2022)
*Z. caespitosa*	Northern	eastern Asia	Vulnerable	Decreasing	Lee et al. (2001)
*Z. caulescens*	Northern	eastern Asia	Near Threatened	Decreasing	Nakaoka & Aioi (2001)
*Z. geojeensis*	Northern	Korean Peninsula	Endangered	Decreasing	Shin, Cho & Oh (2002)
*Z. japonica*	Northern	Asia and invasive in North America	Least Concern	Increasing	Short et al. (2007)
*Z. marina*	Northern	United States, Europe, eastern Asia	Least Concern	Decreasing	Short et al. (2007)
*Z. noltii*	Northern	Europe, north Africa	Least Concern	Decreasing	Short et al. (2007)
*Z. pacifica*	Northern	California Coast, USA	Least Concern	population trend unknown	Obaza et al. (2022)
*Z. capensis*	Southern	coast of Africa	vulnerable	Decreasing	Adams (2016)
*Z. capricornii*	Southern	Australia and New Zealand	Not Listed	N/A	Govaerts (2022)
*Z. chilensis*	Southern	west coast of Chile	Endangered	Decreasing	Jacobs & Les (2009)
*Z. mucronata*	Southern	Australia	Not Listed	N/A	Govaerts (2022)
*Z. muelleri*	Southern	Australia and New Zealand	Least Concern	Stable	Short et al. (2007)
*Z. nigricaulis*	Southern	Australia	Decreasing	Stable	Jacobs & Les (2009)
*Z. novazelandica*	Southern	New Zealand	Not Listed	N/A	Ismail (2002)
*Z. polychlamys*	Southern	Australia	Least Concern	Stable	Jacobs & Les (2009)
*Z. tasmanica*	Southern	Australia	Least Concern	Stable	Les et al. (2002)

With the existence of knowledge gaps and increasing losses for some *Zostera* species, synthesis of knowledge and prioritization of research are essential next steps. Systematic reviews can be effective for consolidating evidence and providing summaries of findings, making them ideal tools for conservation managers aiming to incorporate research in their planning (Centre for Evidence-Based Conservation, 2012). Utilizing decision trees, uniform protocols, and specific guidelines, systematic reviews allow teams of researchers to make unbiased decisions on inclusion and exclusion of articles from a literature search to summarize findings and consolidate data (Clarke, 2011). This consolidation of data can be important for fields where there are data silos, or emerging fields where no centralized conclusions or ideologies have been formed (Lortie, 2014). With seagrass and many other marine ecosystems, systematic reviews are essential for decision making in environmental management and research prioritization, removing the need for every researcher and manager to read through all articles in existence (Ward & Beheshti, 2023). For seagrass specifically, existing reviews and meta-analyses investigated seagrass restoration (van Katwijk et al., 2016; Ward & Beheshti, 2023), seagrass environmental indicators and management (Roca et al., 2016), climate stressor impacts to seagrass (Tang & Hadibarata, 2022), grazer and nutrient effects on seagrasses (Hughes et al., 2004), management thresholds and multiple stressors (Dunic & Côté, 2023), and the cumulative impacts of multiple stressors on seagrass (Stockbridge, Jones & Gillanders, 2020). However, to the best of our knowledge, no previous review studies examined all stressors (studied individually or in combination with other stressors) for all species in one genus of seagrass on a global scale, which is a knowledge gap we aimed to fill.

With this synthesis, we provide a holistic view of stressors and response variables examined, coded according to individual *Zostera* species and geographic locations, thus providing a mechanism for informing the current status of stressor research for seagrasses in the *Zostera* genus and persisting knowledge gaps. Our study sought to lay a centralized foundation of knowledge for seagrass research on stressors, study designs, and response variables *via* a systematic review. By utilizing a very broad definition of stressors, we sought to characterize the many factors (biotic and abiotic) that may influence the marine macrophyte and the ecosystem it supports. The three aims of our systematic review were: (A) to qualitatively assess and summarize the current state of literature focused on seagrass species in the *Zostera* genus and their stressors; (B) to utilize data extracted from full-text articles to identify trends and knowledge gaps for the study of stressor(s), response variable measurements, species, geography, and experimental designs of stressor research; and (C) to map the distribution, type, and number of these studies globally. Our three aims will create the potential to elucidate trends in study of stressors, geography, and *Zostera* species while acting as an open-access tool for informing management and conservation decisions.

## Survey Methodology

### Literature search criteria

We used previous systematic reviews (Dawes et al., 2007; James, Randall & Haddaway, 2016) to develop search elements to find studies on stressors of seagrasses in the genus *Zostera*. H. Lyford and S. J. Bittick performed the Search Strategy. The four key elements were Population/Problem of Interest, Exposure of Interest, Comparator of Interest, and Outcome of Interest (PECO Method) (Morgan et al., 2018). For the current study, the following were identified as key elements: P: Stressor(s) of Seagrass, E: Geographic Area(s), C: Specie(s) of *Zostera*, O: Identification of all stressors present by species and region. Based on key elements and utilizing Boolean Operators, we developed our search terms (Atkinson & Cipriani, 2018). We then input the following finalized terms into the Web of Science (webofscience.com) advanced search: TS = (Zostera* OR eelg* OR seag*) AND TS = (stress* OR temperature* OR nutrient* OR restor* OR reduc* OR ecosys* OR ocean$acid* OR *runoff* OR manipulat* OR conser*) AND TS = (protected* OR phosph* OR nitr* OR nutrient* OR water$quality OR water$temperature OR *growth), Where TS=Topic. We used all databases within Clarivate’s Web of Science (https://www.webofscience.com) in the search, and no initial article filters were incorporated (Clarivate, London, UK). Articles of any language were kept, and later translated if they were included in our review. We included all original research including conference proceedings, review articles, technical reports, dissertations, books, and journal articles in the search stage to allow for an initial search that was as broad and thorough as possible before we excluded any items from our review. This also allowed us to keep a detailed record of all review articles and conference proceedings when they were excluded at the abstract screening step, unless they contained original data. It was important to retain these excluded records for our review, particularly to confirm that a similar review had not already been performed. For our search, it is important to note that the search through Web of Science is not all-encapsulating, as some journals are not indexed by Web of Science (Gates , 2002; Mongeon & Paul-Hus, 2016). We did make efforts to reduce bias in our screening process, however, the use of only one search engine did introduce a level of subjectivity in our methodology towards English language and region-specific journals.

**Figure 1:**
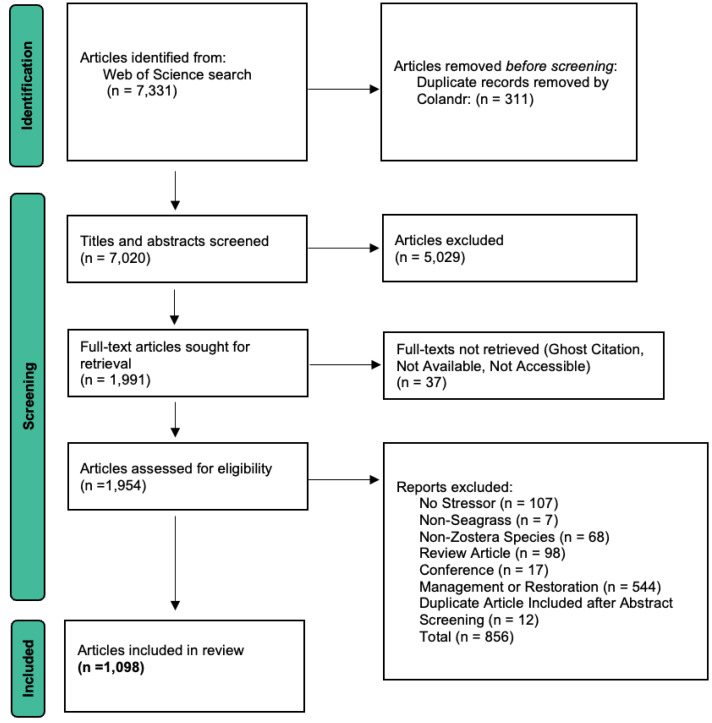
Preferred Reporting Items for Systematic Reviews and Meta-Analyses (PRISMA) flow diagram. Diagram for systematic reviews conducted on database searches. Adapted from Page et al. (2021). Diagram contains study-specific exclusion reasons, duplicates removed during the abstract screening process were removed automatically by ColandrApp (http://www.Colandrapp.com), duplicates removed during full-text screening were removed manually by members of the screening team. The PRISMA 2020 flow diagram template, used here, is from Page et al. (2021). The template is distributed in accordance with the terms of the Creative Commons Attribution (CC BY 4.0) license, which permits distribution, remixing, adaptation and building upon this work, for commercial use, provided the original work is properly cited. To view a copy of this license, visit https://creativecommons.org/licenses/by/4.0/.

We used Colandr (http://www.colandrapp.com) a free, open-source online article screening tool for our systematic screening process (Cheng et al., 2018). Article citations and abstracts were batch-uploaded onto the Colandr platform directly from the Web of Science search as .RIS citations containing the title, abstract, and authors, with 7,331 citations uploaded in total. Duplicate articles were automatically removed by Colandr. The final search and upload occurred on January 10, 2021. We then input all study questions, PECO fields, and added collaborators. Following the set-up process, we established a protocol for article review to narrow down relevant studies. Our protocol was designed following the PRISMA 2020 systematic review checklist (Supplemental Information S1) from Page et al. (2021) and utilized the PRISMA diagram to monitor the flow of the review (Fig. 1). We first screened titles and abstracts, followed by the review and extraction of data from full text articles.

### Follow up searches—species with no articles & other ecosystems

For further investigation of species with no stressor articles, we utilized a similar search methodology in Web of Science as previously listed but added in the species or stressor that was targeted. For example, when searching for *Zostera pacifica* articles, we used the same search terms, adding TS = *Zostera pacifica* * instead of *Zostera.* For the Google Scholar searches of *Heterozostera polychlamys* and *Zostera pacifica* (https://scholar.google.com/), we used species and stressor terms, for example: “*Heterozostera polychlamys*”, “stressor”. We performed additional searches on Web of Science to provide greater context for the number of *Zostera* articles resulting from the screening process, as well as other seagrass genera, corals, and mangroves. These searches were simple topic searches of all databases containing TS = Zostera*, TS = Seagrass*, TS = Coral*, TS = Mangrove*. Publication dates of all articles were obtained directly from the Web of Science database, no additional screening was performed. This was only to visualize article numbers for different ecosystems, but numbers from these searches will be inherently higher than our review numbers.

### Systematic review workflow

#### Title & abstract screening

For title and abstract screening, we developed a methodology for multiple assessors to review articles while minimizing bias and variation (Supplemental Information S2). The review code for initial screening took the form of a decision tree, which reviewers used to screen titles and abstracts and decide whether to include or exclude each article by applying criteria laid out by the tree (Fig. S1). Excluded articles required a reason for exclusion, which was laid out on the decision tree (Fig. S1). Articles included by two reviewers moved on to full-text screening and data extraction. Articles where decisions were in conflict were discussed at a team-wide meeting to reach a resolution. The team leads (H. Lyford and S. J. Bittick) made the final decision on article inclusion or exclusion. Our initial screening resulted in 1,991 articles for our full article screening phase.

#### Full text screening & data extraction

We established the decision tree (Fig. S2), detailed protocol, and training methods (Supplemental Information S3) for full article screening and extraction. Once an abstract was included in full article extraction phase, one screener read each article in its entirety using a second, full-text decision tree to make an include/exclude decision (Fig. S2). Final decisions for excluding an article at this stage were discussed and made by the project leads. We removed duplicate articles that were not automatically removed by Colandr, which resulted in 1,098 articles. Our PRISMA workflow includes the number of all articles originally imported, at which stage articles were excluded, and examples of excluded articles (Fig. 1; Supplemental Information S3). For all included articles, the screener recorded information for all fields in the extraction form (Fig. S3, Data S4.1 and S4.2).

### Study type & study design, publication year

Publication year, study type, and study design of the research were recorded during full-text screening for us to draw conclusions on temporal trends, as well as how most studies were conducted. Year of publication was obtained from each included article’s citation. The types of articles included empirical studies, review articles, technical reports, and model studies if original data were presented. We established study types as model, field, and mesocosm studies, while we divided study designs between survey and manipulative studies. We created detailed definitions for each study type and design to limit variation between screeners (Supplemental Information S4.3).

### Species

We recorded all *Zostera* species studied in each article within the extraction sheet. These were first recorded as the spelling listed by the article and later standardized to the same spelling for data analysis purposes. We used the following as species spellings for data analysis: *angustifolia, asiatica, caespitosa, capensis, capricornii, caulescens, chilensis, geojeensis, japonica, marina, mucronata, muelleri, nigricaulis, noltii, novazelandica, polychlamys, pacifica,* and *tasmanica.* We recorded multiple species per article if the article studied more than one. We did not list additional species outside of the *Zostera* genus from included articles due to the focus of our review.

### Geography

Geography was a consideration in our protocol development, therefore, we required screeners to record the location name, as well as latitude and longitude for each article. If the article only listed the location name, we used Google Earth (Google Earth, version 9.194.0.0, https://earth.google.com/web/) to find the location of the study and obtain coordinates. Additional columns were available for screeners to list up to eight additional locations at the end of the extraction sheet if the article conducted research at more than one site. We used the Polar Geospatial Center Coordinate Converter (Polar Geospatial Center, 2023, https://www.pgc.umn.edu/apps/convert/) to convert coordinates to a standardized format (Decimal Degrees), as needed. Our screeners were not required to list coordinates for model studies or “global” studies. We selected a centralized point for studies with a wide latitudinal range. Coordinates were used to map study type, species, and stressor distribution and density.

### Stressors and response variables

Stressors and response variables were major foci for this systematic review. Emphasizing a very broad definition, we defined stressors in this study as anything abiotic (*e.g.*, hydrodynamics, light, temperature) or biotic (*e.g.*, herbivory, seagrass competition, epiphytes) studied by the article as having an influence on the *Zostera* seagrass of interest. Note that the impact of the stressor studied is not always negative but, in some cases, exists across a gradient (*e.g.*, light, temperature, or nutrients). We compiled a list of stressors from our reading during abstract screening, also allowing article screeners to suggest stressors encountered further into the review process, resulting in 23 possible stressors (Fig. S3), and we provided descriptions and examples to screeners during full article data extraction (Supplemental Information S5). Additionally, to further characterize our definition of stressors, we grouped them into “umbrella categories” from Kappel (2005)’s list of marine threats. These categories included pollution (*e.g.*, nutrients, sediment), climate change (*e.g.*, temperature, drought) increased anthropogenic presence (*e.g.*, aquaculture impacts, anthropogenic use), and intrinsic factors (*e.g.*, herbivory, pathogens) to analyze trends in category frequency and stressors studied together (Supplemental Information S5).

For umbrella category analysis, we counted and summed full-text articles that recorded stressors under each of the four unique categories. Since each article could have stressors under multiple umbrella categories, and thus be counted twice, our total article numbers across all umbrella categories exceeded the full-text article count. Articles that recorded multiple stressors under the same umbrella category were not double counted. For our calculation of the most frequently recorded species and stressors used for the creation of a heat map, we normalized the frequency of combinations of stressors and species of *Zostera*. Normalization was performed by summing all articles that studied each stressor for each species, then dividing by the total number of articles (regardless of stressor) that examined that species to get a percentage value. Since each article could investigate more than one stressor, the sum of the percentages within each column (*e.g.*, for each species) could exceed 100%. Percentages calculated were relative to each species, but normalization was scaled for heat map coloration from the lowest possible percentage (0%) to the highest possible percentage of articles (64%) for all species. Note we only included the top 10 studied stressors as indicated by counts of full-text articles that recorded each individual stressor, and we only included eight species of *Zostera* with greater than 14 articles based on counts of the most frequently recorded species in full-text articles.

In addition to stressors, individual response variables were classified within three categories based on levels of biological organization. These categories included plant (individual), community (biotic aspects of seagrass ecosystems), and environmental (abiotic aspects of seagrass ecosystems) (Fig. S3) with different individual response variables recorded in each of the three categories. We defined the response variables as aspects of the study measured in response to stressors, in categories associated with the seagrass, community, and/or environment. Each screener could list up to four response variables within each category, but population of all fields in each category was not required. Additional response variables could be listed in later extraction form columns if the number exceeded four. Based on these categories, we described each response variable measurement with examples and citations for project member reference during full-text screening (Supplemental Information S6).

### Data analysis and visualization

We performed visualizations of frequency, overlap, and associations using GraphPad Prism (GraphPad Software, La Jolla, CA, USA) for study type and study design, species, geography, stressors, publication year, and response variables. Our frequency analysis examined the number of articles recorded per study type and study design, species, geography by country, response variables by category, stressors, and publication years. We aimed for this analysis to provide insights on the frequency and overlap of study for study type and study design, species, geography, stressors, publication year, and response variables. Finally, we kept detailed records of articles excluded at each phase of screening, as well as any articles removed during the data analysis phase (Data S7).

## Results

Following title and abstract screening, we included 1,098 full-text articles for our data extraction and analysis. From these 1,098 articles, data were generated for the following overarching topics: study type and study design, publication year, species, stressors, response variables, and geography to summarize the current state of research focused on the *Zostera* genus.

### Study type and study design

**Figure 2:**
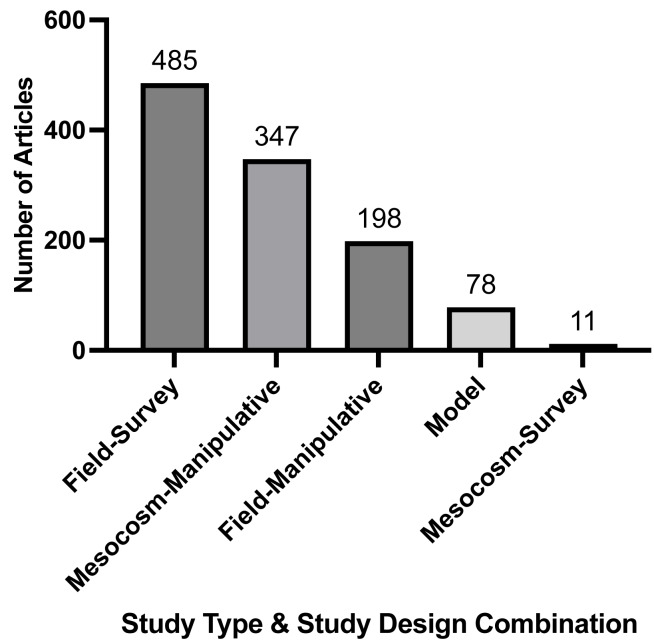
Frequency of study type and study design combinations recorded by full-text articles. Study type included field, mesocosm, and model studies. Study design included survey and manipulative studies. Numbers may be greater than the total number of articles due to models tagged in conjunction with other study types and study designs.

We found the most common type and design of included research articles were field-survey studies (*n* = 485, Fig. 2). There were a number of model studies (*n* = 78, Fig. 2) but they were much less frequent than field and mesocosm studies. Overall, field-survey studies had a greater portion of the total articles measuring three to four or more stressors compared to mesocosm-manipulative studies (Table 2). However, the majority of articles for both field-survey and mesocosm-manipulative studies measured one or two stressors (Table 2). Conversely, model studies and technical reports had the highest percent of their respective total articles measuring two or more stressors (Table 2). Looking at studies over time, the general trend shows all study types and designs increasing; however, there is a sharper increase in model studies around the year 2000 as compared to zero studies for almost all years prior (Fig. S4).

### Publication year

**Figure 3:**
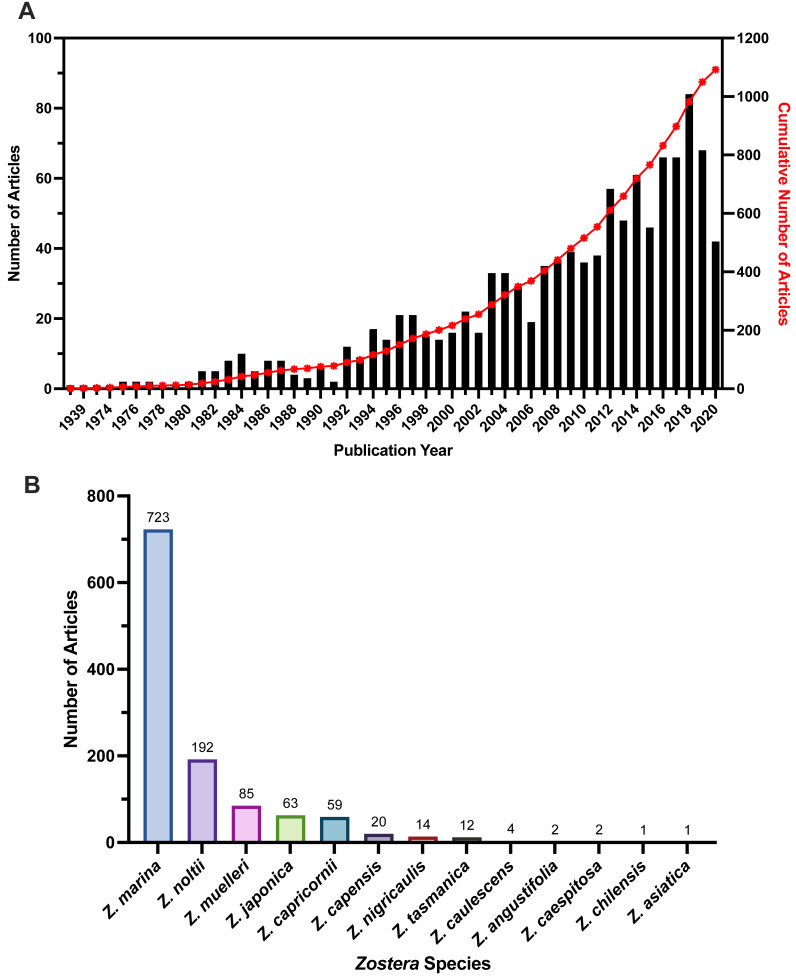
Publication year and species numbers for full-text articles included in the review. (A) Number of articles per publication year for included full-text articles, displayed on left *y*-axis. Cumulative individual publication year as a percent of the total articles displayed on the right *y*-axis. Many of the 2021 publications were pre-prints at the time of literature search, and article numbers were not yet representative of 2020 and 2021. Data for years 1938–2020 are shown. (B) Frequency of *Zostera* species in included full-texts. Articles could have multiple species listed, which is why the total overall numbers for all species in this figure exceeds the total article number for our review. *Zostera* species with 0 articles not shown.

We found an increasing number of articles published in recent years (Fig. 3A), with the greatest number of articles published in 2018 (*n* = 84 articles). The earliest published article was 1938, and the most recent article was published in 2021 at the time we finalized our literature search in January of 2021. Most 2021 publications were pre-prints at the time of literature search, and article numbers are only representative up until the date of the search execution, meaning the 2020 and 2021 numbers may not be completely represented by our review. Compared to overall articles published per year from Web of Science for the *Zostera* genus, all seagrasses, and two other coastal ecosystems (corals and mangroves), coral reefs consistently had the highest number of articles published. Seagrasses were the least frequent of the three coastal ecosystems (coral reefs, mangroves, and seagrasses) the articles included in this study had the lowest number per year overall (Fig. S5).

**Table 2:** The number of full-text articles based on the number of stressors recorded for each study type and study design. The number of articles (percentage of total articles in each column for that type/design in parenthesis) based on the number of stressors recorded for study types (field, mesocosm), study designs (survey, manipulative), as well as model, technical report, and review studies.

**Number of stressors recorded**	**Field-survey**	**Mesocosm- manipulative**	**Field- manipulative**	**Model**	**Mesocosm- survey**	**Review**	**Technical report**	**Total number of articles per stressor**
1 Stressor	158 (32.6%)	142 (40.9%)	76 (38.4%)	10 (12.8%)	5 (50.0%)	3 (50.0%)	1 (20.0%)	395
2 Stressors	159 (32.8%)	145 (41.8%)	70 (35.4%)	21 (26.9%)	4 (40.0%)	2 (33.3%)	2 (40.0%)	403
3 Stressors	110 (22.7%)	50 (14.4%)	41 (20.7%)	27 (34.6%)	1 (10.0%)	1 (16.7%)	0	230
4+ Stressors	58 (12.0%)	10 (2.9%)	11 (5.6%)	20 (25.6%)	0	0	2 (40.0%)	101
**Total number of articles per study type/study design**	485	347	198	78	10	6	5	

### Species

Our data extraction indicated inconsistencies in the naming of species within the genus *Zostera,* and an expansion of the nine species previously identified in Short et al. (2007) and Olsen, Coyer & Chesney (2014). Along with these nine species (*Z. asiatica*, *Z. capensis, Z. capricornii, Z. japonica, Z. marina, Z. muelleri, Z. nigricaulis, Z. noltii,* and *Z. tasmanica*) we recorded inconsistencies from paper to paper in the spelling of the following species: *Z. nolti vs. Z. noltii; Z. capricorni vs Z. capricornii* vs. *Z. capricornia;* and *Z. mulleri vs. Z. muelleri.* However, beyond the spelling differences between articles, we recorded more than nine species during our review for the *Zostera* genus. Sources now list up to 18 species within the *Zostera* genus (Govaerts, 2022, Table 1). However, names and numbers have fluctuated, particularly following the nesting of other genera (*Heterozostera, Zosterella*, and *Nanozostera*) within the *Zostera* genus (Jacobs & Les, 2009; Coyer et al., 2013; Sullivan & Short, 2023). Our data extraction resulted in 13 total species (Fig. 3B), which is four more than the original nine described in the hallmark Short et al. (2007) article. The additional species we recorded were *Z. angustifolia, Z.* c*aespitosa, Z. caulescens,* and *Z. chilensis.* However, even our findings differed from and fell short of the 18 species described by other researchers (Govaerts, 2022), which also includes *Z. geojeensis*, *Z. mucronata, Z. novazelandica*, *Z. pacifica*, and *Z. polychlamys*. When we examined why no articles were found for *Z. polychlamys*, re-running the literature search on Web of Science with *Heterozostera polychlamys* returned only three papers, searching Google Scholar for the terms “*Heterozostera polychlamys”* and “stressors” returned one paper, and searching Google Scholar for just “*Heterozostera polychlamys”* resulted in 15 papers. To further understand the low article numbers relating to *Zostera pacifica,* we re-ran our Web of Science search, replacing “*Zostera*” with “*Zostera pacifica*” which returned eight papers, and searched Google Scholar for “*Zostera pacifica”,”* stressor*”* which returned five papers. For these papers, four were published following the conclusion of our search, the final paper was a review paper.

We found the most frequently studied species in the *Zostera* genus was *Zostera marina* (*n* = 722, Fig. 3B). Some articles studied multiple species, with most overlap occurring between *Z. marina* and *Z. noltii* (*n* = 42), as well as *Z. marina* and *Z. japonica* (*n* = 23). Only three studies examined three different species: these were *Z. angustifolia, Z. marina*, and *Z. noltii* (Lima, Ward & Joyce, 2020)*, Z. caespitosa, Z. japonica,* and *Z. marina* (Namba & Nakaoka, 2018), and *Z. marina, Z. muelleri,* and *Z. tasmanica* (Mazarrasa et al., 2019). Most studies focused on a single species (*n* = 1, 009). Two species, *Z. asiatica* and *Z. chilensis*, had only one article each that listed them as the species of interest (Park, Kim & Park, 2016; Sandoval & Edding, 2015). Altogether, these results show a high interest in single species studies for this genus, with a high frequency of study for *Z. marina* in the included full-text articles.

### Geography

**Figure 4:**
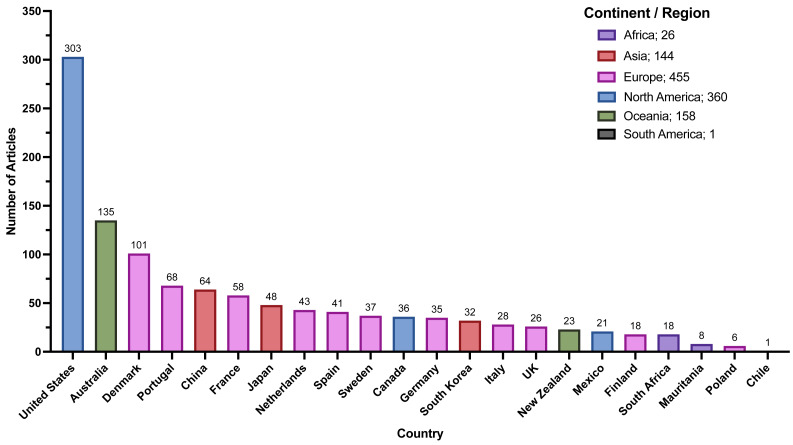
Article numbers per country recorded during full-text extraction. Number of articles recorded for each country. Colors of bars are associated with continents/regions where purple is Africa, red is Asia, pink is Europe, green is Oceania, blue is North America, and grey is South America. Only countries with greater than five articles are displayed aside from one included article from South America, specifically in Chile.

The most common locations for *Zostera* studies from our review by continent/region were North America, Europe, and Oceania (Fig. 4). For countries, similar trends arose with the United States of America (USA), Australia, Denmark, and Portugal producing the bulk of these articles (Fig. 4).

**Figure 5:**
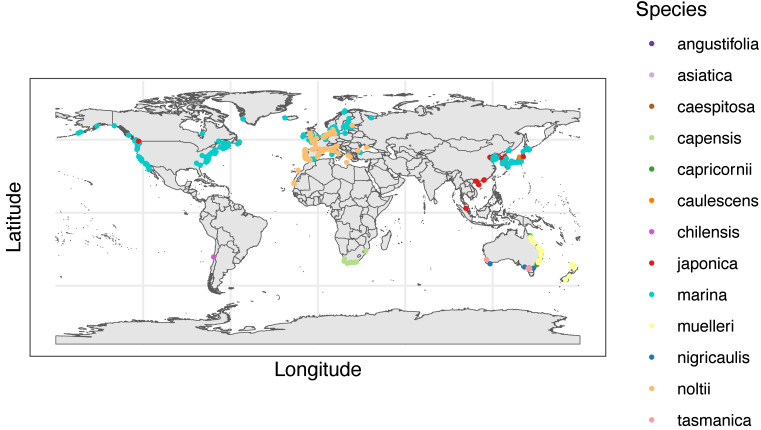
World map of *Zostera* species recorded in full-text articles. World map with dots representing the location(s) of each full text article. The colors of the dots correspond to 13 species of *Zostera*. Made with Natural Earth. Free vector and raster map data @ naturalearthdata.com.

When we analyzed species distributions by geography (Fig. 5), it is important to point out that although there were hotspots of study, these hotspots are not necessarily indicative of greater *Zostera* presence, but rather research effort identified by our search towards the study of *Zostera* seagrass in a certain location. *Zostera marina* was not only the most frequently studied species in all of our included articles, but it also had the widest geographical distribution of studies (Fig. 5). Some continents with known *Zostera* populations lacked uniform distributions of their studies (Fig. 5). One case of this exists within North America, where we found studies were largely concentrated on the east coast of the USA with a subset on the west coast, while Canada and Mexico, countries with known *Zostera* seagrass populations, had relatively few articles cited throughout each country (Fig. 5). In addition, we found there were places that have *Zostera* seagrass populations, but smaller article numbers were returned by our review’s search, particularly South America and Africa (Fig. 4). For South America, our literature review only returned one article, which was from Chile. To allow users to access data and articles from our review, we created an interactive map from our systematic review data (Google Map of Systematic Review), where all locations of studies as well as their metadata can be accessed by interested users to ameliorate confusion in species and study geography. We performed our search and review additional times on 5/16/2023 and 5/24/2024, which we intend to repeat annually in May, with ongoing screening occurring. Members of the Bittick Lab at Loyola Marymount University have screened abstracts through the 5/24/2024 search and are presently extracting data from the 749 new articles. We will periodically update our interactive map with this additional data from recent years.

### Stressors

**Figure 6:**
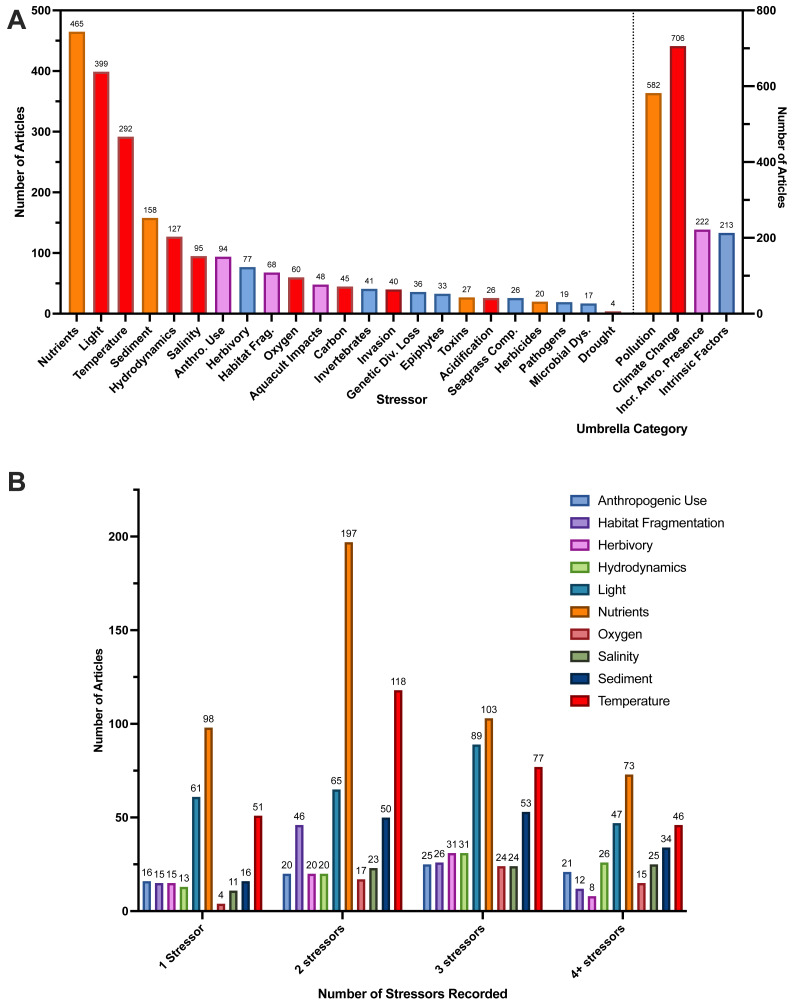
Number of articles that recorded each of the 23 individual stressors with *Zostera* species and breakdown of stressors. (A) Number of full-text articles that recorded each of the 23 individual stressors (left *y*-axis) and the number of full-text articles that recorded stressors under each of the umbrella categories (right *y*-axis). Individual stressors are colored corresponding to the umbrella categories shown on the right *y*-axis (Pollution, Climate Change, Increased Anthropogenic Presence, Intrinsic Factors). Since each article could have stressors under multiple umbrella categories, total full-text article numbers across all umbrella categories exceeds full-text article count. Articles that recorded multiple stressors under the same umbrella category were not double counted. These umbrella categories and their associated stressors are described in-depth in Supplemental Information 5. Abbreviations are as follows: Anthro. Use (Anthropogenic Use), Gen. Diversity (Genetic Diversity), Habitat Frag. (Habitat Fragmentation), Aquacult. Impacts (Aquaculture Impacts), Seagrass Comp. (Seagrass Competition). (B) Top 10 most frequently studied stressors, broken down by the total number of stressors recorded per article. Full-text articles were first grouped based on the total number of stressors recorded (1, 2, 3, 4+), then a count of each individual stressor was calculated within each group to obtain the number of articles. Since each article could investigate more than one stressor, the sum of all bars (*e.g.*, for each stressor) can exceed the total article number (*e.g.*, in the two-stressor group, the bar for nutrients (*n* = 197), is displaying that nutrients were recorded 197 times by articles that measured two stressors. The 10 most frequent stressors used were determined by the highest overall article counts shown in (A).

At the conclusion of article screening, we extracted data from full-texts across the 23 types of stressors we described (Supplemental Information S5). The most frequently examined stressor in articles was nutrients, followed by light and temperature (Fig. 6A). These three stressors also had the greatest frequencies for articles that recorded three and four total stressors (Fig. 6B). Most articles studied two stressors (*n* = 403 articles), followed by a single stressor (*n* = 395 articles) (Table 2). When we look at patterns in the number of stressors that articles recorded over time, articles measuring more than one stressor were consistently higher than those that measured only one stressor after the year 2000 (Fig. S6A). Furthermore, when broken down by one, two, three, and four or more stressors measured, articles measuring one and two stressors were the most prevalent in our data, articles measuring three stressors increased in frequency after the year 2000 (Fig. S6B). Finally, in our analysis of umbrella categories, we found the greatest number of articles recorded stressors categorized under the climate change umbrella, followed by pollution, then increased anthropogenic presence, and finally intrinsic factors (Fig. 6A, Supplemental Information S5). Furthermore, we categorized 77% of stressors as pollution and climate change-related (Fig. 6A). For our analysis of individual stressors measured by publication year, many follow similar patterns of increases in study over time, similar to publication year alone. However, some sharper increases were observed for anthropogenic use, habitat fragmentation, and sediment after the year 2000 (Fig. S7).

**Figure 7:**
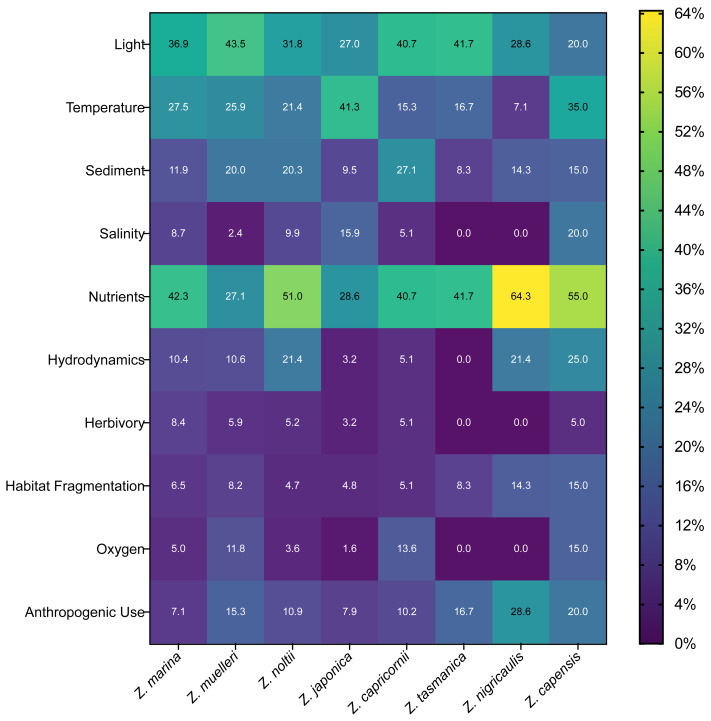
Frequency of occurrence for top ten stressors and top eight species in full-text articles. Heat map showing the normalized frequency of combinations of stressor and species of *Zostera*. Numbers within the heatmap indicate the percent of articles in our review that studied each stressor for each of these eight *Zostera* species. Percentages in each column are relative to each species, but the heatmap color is scaled from the lowest possible percentage (0%) to the highest possible percentage of articles (64%) for all species. Normalization was performed by summing all articles that studied each stressor for each species, then dividing by the total number of articles (regardless of stressor) that examined that species. Since each article could investigate more than one stressor, the sum of the percentages within each column (*e.g.*, for each species) can exceed 100%. The total number of articles that studied each of these top eight seagrass species were *Z. marina*: 722, *Z. muelleri*: 85, *Z. noltii*: 194, *Z. japonica*: 64, *Z. capricornii*: 59, *Z. tasmanica*: 12, *Z. nigricaulis*: 14, and *Z. capensis*: 20. Note we only included the top ten studied stressors as indicated by counts of articles that recorded each individual stressor, and we only included eight species of *Zostera* with greater than 14 articles based on counts of articles that recorded each species.

Across *Zostera* species, we found light, nutrients, temperature, and sediment were the stressors studied most often for most species, comprising 63% of these species’ total stressors recorded. *Zostera nigricaulis* had a strong level of occurrence with nutrients, and it was also the species studied most with anthropogenic use, rather than temperature (Fig. 7). Interestingly, but perhaps unsurprising, there is a common association of *Zostera* species with the study of stressors that we categorized under the umbrella categories of pollution or climate change (Supplemental Information S5, Fig. 7). These stressor frequencies did vary slightly by species; however, nutrients and light were a common point of study for the eight most frequently studied *Zostera* species (Fig. 7). However, *Z. capensis*, *Z. muelleri, Z. nigricaulis*, and *Z. tasmanica* did have greater than 23.5% of their respective total articles measuring more than one stressor in the increased anthropogenic presence umbrella category, all other species fell under 15% (Fig. 7).

### Response variables

**Figure 8:**
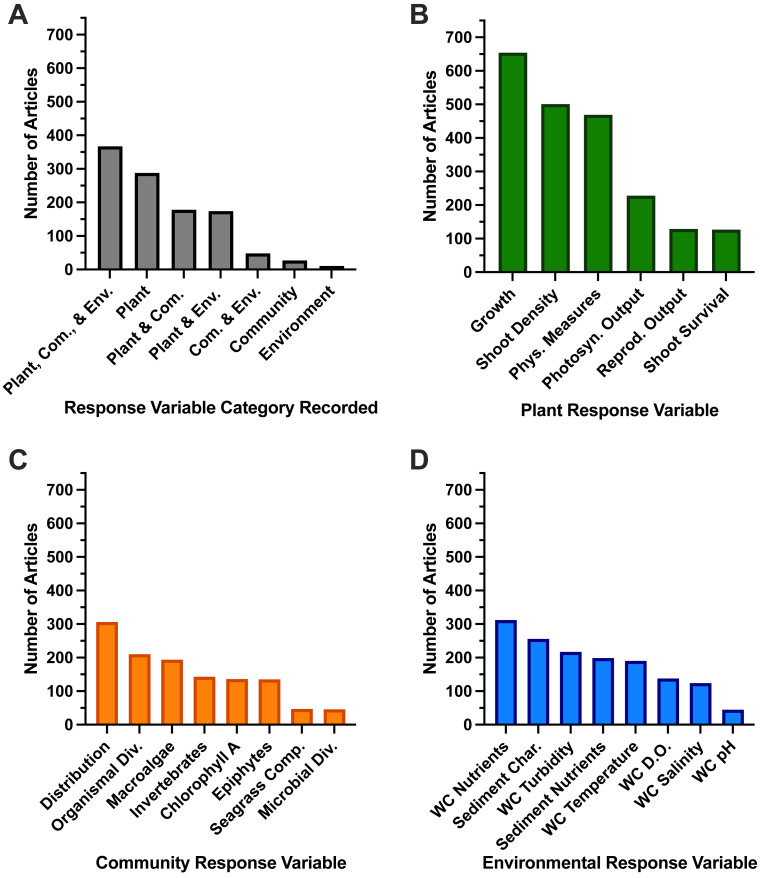
Number of full-text articles that investigated combinations of response variable categories. (A) The number of full-text articles that investigated combinations of response variable categories. The number of full texts that investigated each individual response variable, which we categorized as (B) Plant Response Variables, (C) Community Response Variables, or (D) Environmental Response Variables. Abbreviations we used are as follows: Plant: Phys. Measures (Physiological Measures), Photosyn. Output (Photosynthetic Output), Reprod. Output (Reproductive Output). Community: Organismal/Microbial Div. (Diversity), Seagrass Comp. (Seagrass Competition). Environment: WC (Water Column), Char. (Characteristics), DO (Dissolved Oxygen).

The greatest number of articles (*n* = 367, Fig. 8A) recorded response variables in all three of our categories (plant, community, and environment), followed by articles that only recorded plant response variables (*n* = 288 of total articles, Fig. 8A). Looking at individual response variables, “growth” was measured most often in 59.5% of studies (Fig. 8B). Other common individual response variables and percentage of total studies recorded include “shoot density” (45.6%), “physiological measures” (42.7%), “water column nutrients” (28.4%), “distribution” (27.9%), and “sediment characteristics” (23.3%) (Figs. 8B–8D). When research studies recorded response variables across two or three categories, “growth,” “shoot density,” “water column nutrients,” “physiological measures,” and “distribution” were still often the most abundant individual response variables (Fig. S8).

## Discussion

This systematic review extracted data from 1,098 articles, surpassing the scopes of many other systematic reviews of seagrass, and creating a foundational piece of knowledge and map for researchers moving forward. Many systematic reviews have examined specific case studies (Griffiths, Connolly & Brown, 2020; Kendrick et al., 2019), reviewed restoration articles (van Katwijk et al., 2016; Ward & Beheshti, 2023), or summarized meadow structure, biomass, and production (Strydom et al., 2023). All these pieces of work are foundational to seagrass research, but for our specific aims of summarizing work for the *Zostera* genus, few have included the number of articles, breadth of geography, or number of species within a single genus that our systematic review describes. Broad geographical coverage in our study elucidated trends in stressors, response variables, and species by study location, which serve impactful on a global scale, as data can be viewed on many levels of geographical area and boundaries. Our search produced extensive data, but possible data limitations include the use of one database (Web of Science), inability to access some articles, and the exclusion of unpublished data. The main findings from our study point to trends in the frequent study of certain stressors, particularly nutrients, temperature, and light; *Z. marina* as the species with the highest study numbers for the review, with other species missing entirely from our included articles; and disparities in research occurrences by geographic location. From the data we synthesized, we identify the main knowledge gaps and discuss the significance of study trends for future data use, and to inform future research and management efforts in our discussion.

### Study designs for seagrass research have seen a growing diversification of methods over time and will continue to benefit from different and emerging study designs

We found field-survey studies were the most common, likely due to an ability to observe *in situ* conditions while meeting the needs for monitoring and generating distribution data for *Zostera* seagrasses (Sousa et al., 2019; Ostrowski et al., 2023). Additionally, high numbers of field-survey studies may be attributed to difficulties faced by both manipulative-field and mesocosm studies in altering multiple stressors at once, or in mesocosms’ ability to produce results comparable to real world conditions (Falkenberg, Russell & Connell, 2016). However, experimental manipulations have merit to understand how stressors impact seagrasses, and which mitigation techniques prove most successful.

Mesocosm-manipulative studies may face obstacles of *in situ* applicability and high equipment needs to meet replication requirements for multiple stressor studies. However, they do allow experimenters to reduce confounding variables, and offer a method for simulation of varying scenarios, making them a powerful design (Blake & Duffy, 2010; Stewart et al., 2013). Moreover, recent technology has increased the ability of mesocosms to replicate real-world conditions, which has been well-refined for ocean acidification experiments (Wahl et al., 2020). Advancements in mesocosms could be applied to diversify *Zostera* seagrass research study designs without significant applicability drawbacks. In addition to mesocosms, studies might consider integrating a modelling approach with a mesocosm experiment to enhance the model simulations with parameters derived from the mesocosm (Ullah et al., 2020). From our own collation of data, we did begin to see increases in the use of modeling as a study design in recent years, which is a positive step towards integrating diverse methods for a robust understanding of *Zostera* seagrasses. In the realm of multiple stressors and seagrass research, these designs could profoundly impact our ability to experiment, model, and predict certain stressors and responses for the *Zostera* genus (Adams et al., 2020; Jakobsson-Thor et al., 2020). Other novel technologies include drone mapping, remote sensing, and environmental DNA metabarcoding, which show promise in their ability to monitor and understand field conditions and seagrass ecosystems long-term (Marvin et al., 2016; Beng & Corlett, 2020). As these methods become more widely used and available in marine science, they can provide us with more feasible methods for monitoring remote areas or locations without distribution data (Duffy et al., 2019; Clemente, Thomsen & Zimmerman, 2023).

Indigenous Knowledge and local communities can be assets to management and baseline information needs. Therefore, they should be recognized and consulted, or studies co-developed, whenever possible (Brondízio et al., 2021; Loch & Riechers, 2021). Some examples of Indigenous involvement occur in Australia, where Indigenous Communities have been heavily involved in mapping habitat extent and managing seagrasses (Seagrass Watch), as well as successes in co-developing marine protected areas and managing fisheries alongside Indigenous Peoples (Hall-Arber, Pomeroy & Conway, 2009; McKenzie et al., 2014; Strickland-Munro et al., 2016). Other examples include citizen science efforts with local communities, such as “SeagrassSpotter”, an online tool and app for users to submit data on seagrass distributions, health, and status (Jones et al., 2016). Seagrasses hold significance to many Indigenous Peoples and local communities; however Indigenous and local knowledge is rarely included in the conservation discipline (Jones et al., 2024). One recent study found that despite the widespread nature and high value that Indigenous Knowledge holds for long-term monitoring and local stewardship of seagrass ecosystems, few seagrass management plans co-produce knowledge or integrate rights and local resource use for Indigenous Communities (McKenzie et al., 2021). Presently, *Zostera* research would benefit from deeper connections and collaboration with Indigenous Peoples and local communities to generate a more inclusive understanding of these social-ecological seagrass systems, and fill persistent knowledge gaps (Hill et al., 2020; Jones et al., 2022). Overall, given the current state of study designs and study types, our review shows a positive outlook for the field’s increasing ability to understand *Zostera* seagrass biology and ecology *via* the integration of novel technology with traditional methods, consulting and collaborating with Indigenous Peoples and local communities, and exploring advancements in our ability to perform realistic mesocosm or field-manipulative experiments.

### Response variables would benefit from continued use and greater standardization for the *Zostera* genus

The majority of included studies recorded response variables in all categories (plant, community, environment). This may point to studies considering both the status of seagrass plants in addition to associated abiotic and biotic variables, which aligns with our review’s focus on stressors studied with *Zostera* seagrasses. These measured variables also provide important data on individual plant and community- to ecosystem-wide health (Roca et al., 2016). Our synthesis of variables that were measured is informative for experimental design and selection of indices for monitoring or measuring management success (Wood & Lavery, 2000). The most commonly measured individual response variables, which were growth, shoot density, physiological measures, water column nutrients, distribution, and sediment characteristics, would benefit from continued reference when designing future studies for this genus. For *Zostera* in particular, growth metrics can be important indicators for light limitation, where shoot density and biomass have been used as indicators for *Z. marina*, *Z. japonica*, and *Z. noltii* to assess overall meadow health (Huong et al., 2003; Bertelli & Unsworth, 2018; Vieira, Lopes & Creed, 2018). Past study of seagrass and monitoring indicators found many indicators were spatially and temporally limited, with little consensus on the variables/indicators measured, if they are measured at all (Unsworth et al., 2018). Further, differences in the methodology of response variables measured can hinder comparisons of data across studies or regions, impeding the ability to scale results (Nelson, 2017). Our finding that many studies focused on similar response variables indicates a positive movement in *Zostera* seagrass-stressor research towards a consensus on types of variables that should be measured for this genus. Therefore, we encourage continued future use of these response variables and new studies of *Zostera* species to integrate response variables appropriately.

To assist with this, our results can be further interrogated on Open Science Framework (Open Science Framework) and Google Maps (Google Map of Systematic Review), *via* an interactive map of the review data. On OSF, a link to our interactive global map of article titles, species, stressors, study type and study designs, and response variables is featured to provide reference for future *Zostera* studies. Furthermore, measuring the same variables in the same manner will allow for comparable data across multiple studies, lending to collaboration and research connectivity within the *Zostera* field. Repeated measurement of similar variables fits with the ideas presented by Fraser et al. (2013), where coordinating distributed experiments can answer a similar research question for a range of locations and reduce site differences for meta-analyses and collaboration. *Zostera* Experimental Network (http://zenscience.org/), SeagrassNet (https://www.seagrassnet.org/), Seagrass Watch (https://www.seagrasswatch.org/), Seagrass Trait Database (https://seagrasses.ccmar.ualg.pt/), and The Marine Global Earth Observatory (https://marinegeo.si.edu/protocols/seagrass-habitats) have made major strides towards achieving goals of standardized methods and mapping seagrass distributions (McKenzie et al., 2000; Duffy et al., 2015; Short, Coles & Short, 2015; de los Santos et al., 2022, and Lefcheck, 2022). These efforts will be particularly relevant for ameliorating past challenges pointed out by Unsworth et al. (2018) with indicators, in addition to tackling some of the knowledge gaps identified by this review, particularly with regard to data centralization.

### *Zostera* taxonomic assignment and study of species varies widely with little centralized agreement

The lack of centralization regarding species of seagrass in the *Zostera* genus was unexpected with regards to the degree of variation in spelling and taxonomy. For *Zostera,* one of the most diverse and prolific seagrass genera, little understanding and centralized agreement of species holds high implications for the genus. Many articles in our review had different spellings of certain species, while other articles listed species that are only recognized as subspecies by others (Short et al., 2011; Kuo et al., 2018). These findings point to an issue of little consensus within the *Zostera* genus. In reviewing papers focused on *Zostera*, there is a general pattern of a growing number of defined species subsequent to Short et al. (2007), which only defined nine species. Growing interest in seagrass conservation and resulting increased research effort has encouraged closer inspection of these originally defined species; however, this seems to leave taxonomic definition in flux. With recent advances in whole genome sequencing, part of this growing number results from genetic sequencing indicating that some seagrass species contained multiple species that are phenotypically similar, such as *Z. marina* and *Z. pacifica* (Coyer et al., 2013). Because of these findings, we expect that as seagrass research continues to converge with the field of molecular biology, fluctuation of defined *Zostera* species numbers is likely to occur (Sullivan & Short, 2023). Our own results grew this number to 13, however, we still recorded no articles for five of the species described by other researchers (*Z. geojeensis*, *Z. mucronata, Z. novazelandica*, *Z. pacifica*, and *Z. polychlamys*). Although some variance will persist, the interactive map featured on our OSF will allow users to view the study, species, and geographic location that we recorded for every article to provide some clarity on this issue. Each pin on the map is color-coded by species so users can select articles by species and view publication years to better inform how they can name and identify species. The species-taxonomic knowledge gap that persists for *Zostera* is an important one, as variation within species is a powerful consideration for researchers and managers.

Upon further investigation of the five species missing from articles in our review (*Z. geojeensis*, *Z. mucronata, Z. novazelandica*, *Z. pacifica*, and *Z. polychlamys*), *Z. mucronata* and *Z. novazelandica* are now regarded as subspecies of *Z. muelleri*, and *Z. polychlamys* was formerly *Heterozostera polychlamys* which may explain some of the lack of data for these species (Kuo et al., 2018 and Larkum, Waycott & Conran, 2018). Despite these explanations, *Z. pacifica* and *Z. polychlamys* are listed as accepted species within the *Zostera* genus in the Global Biodiversity Information Facility (GBIF) backbone taxonomy database (Govaerts, 2022; GBIF Secretariat, 2023). On the other hand, *Z. geojeensis* is listed as a species by the IUCN Red List but is not regarded as its own species according to the GBIF backbone taxonomy database (Govaerts, 2022; IUCN, 2022). Researchers must have a fundamental understanding of a specific species to reference past literature and correctly share findings, which managers may use for their efforts in conservation. Without centralized agreement, differences between species may be overlooked (de los Santos et al., 2016). Regardless, it may be pertinent for future studies to include and reference all naming conventions in their research for these five species.

Since *Z. geojeensis*, *Z. pacifica*, and *Z. polychlamys* are all identified as species by various sources, we aimed to identify where some of these discrepancies originated. For *Z. polychlamys*, there is debate about whether the genus *Heterozostera* should be a subgenus of *Zostera*, resulting in different names and classifications for this species (Jacobs & Les, 2009). Since this 2009 article, other articles have used both *Zostera polychlamys* and *Heterozostera polychlamys* (Borum et al., 2016; Sullivan & Short, 2023). Therefore, little taxonomic consensus seems to exist for the nesting of this genus, causing difficulties determining total article counts for this species, and understanding how or if the species is impacted by stressors. For *Z. pacifica,* the primary justification for its low article numbers is its historical categorization as *Z. asiatica* or *Z. marina* (Coyer et al., 2008). Thus, some of the articles studying *Z. asiatica* or *Z. marina* on the west coast of the USA may also have been incidentally studying *Z. pacifica*. Unfortunately, there is very little ability to retroactively sort out which articles may have done this; thus, *Z. pacifica* remains an under-studied species. This problem stretches into a space of larger public awareness as well: NOAA’s informational website on west-coast seagrasses in the USA omits *Z. pacifica* as a species, defining *Z. marina* as the only species of eelgrass present in the area (NOAA Fisheries, 2022). Finally, there is similar taxonomic debate for *Z. geojeensis*, as conflicting sources list it as its own species, or a sub-species of *Z. caespitosa* (Short et al., 2011; Yoon & Kim, 2021). Nevertheless, *Z. geojeensis* is listed as Endangered by the IUCN Red List, and this taxonomic perplexity and lack of information could be harmful to threat assessments and conservation efforts for the species (Short et al., 2010). Although we can explain some part of the deficit in article numbers for *Z. pacifica*, *Z. polychlamys*, and *Z. geojeensis,* having three *Zostera* species with zero articles remains a large knowledge gap from our review. Increasing taxonomic consensus for the naming of *Zostera* species will be important to accurately understand their status, distribution, and species-level threat and stressor assessments (Mounce et al., 2018). Therefore, we recommend that future research studies reference the most recent taxonomic review from Sullivan & Short (2023) when describing *Zostera* and *Heterozostera* species.

Our review indicated that the majority of research articles focused on *Z. marina* (722 articles), far exceeding the number of articles recorded for the second most frequent species, *Z. noltii* (194 articles). This was not initially surprising because of *Z. marina*’s extensive range and establishment as the most-studied species over the longest period of time; however, the magnitude of difference was unanticipated (Moore & Short, 2006). Although it is encouraging to see the research effort for this species, it seems the *Zostera* genus is considered well-studied overall due to the volume of research on *Z. marina* alone (Moore & Short, 2006; Nordlund et al., 2016). Three challenges arise from this: (1) *Zostera* species have high phenotypic plasticity, therefore knowledge of one species is not always universally applicable; (2) the recent characterization of *Z. pacifica* presents issues with separating research findings from *Z. marina* prior to the name change; and (3) for those in restoration, it will be pertinent to consider the species identity and genetic diversity as projects with specific species (*Z. pacifica*) or high genetic diversity have been shown to have greater success (Coyer et al., 2008; van Katwijk et al., 2016; Pazzaglia et al., 2021). Dismantling the assumption that data for *Z. marina* is sufficient for all species in the genus will help to close gaps in knowledge and improve species-specific restoration and management decisions.

There is fewer comprehensive data on seagrass distributions outside of *Z. marina.* In Short et al. (2007)’s list of species present in different bioregions (delineated by the paper based on species assemblages, distributional ranges, and tropical/temperate zones), *Z. capensis, Z. japonica,* and *Z. noltii* are the second most widely distributed species. All were present in two bioregions each *versus* the three that contain *Z. marina.* Comparing this to our findings, we found *Z. noltii* had the second most number of articles, while *Z. japonica* and *Z. capensis* had lower article numbers than expected given their larger distributions. Many of the countries contributing to the article numbers of *Z. noltii,* and even the third most studied species, *Z. muelleri,* were high-income countries, including Australia and many European countries. On the contrary, the countries where *Z. japonica* and *Z. capensis* are found include many in the South Pacific, and for *Z. capensis*, countries in Africa, many of which are classified as low- and middle-income countries. With these results, we saw a stark trend of lower data availability and research in low- and middle-income countries, similar to findings from Nordlund et al. (2016). While this finding is not novel, we highlight our observation of the specific species exhibiting this trend, since the previous study looked at inter-genus related differences. *Zostera japonica* and *Z. capensis* are both located in low- and middle-income countries, and given their lower article numbers in this review, this may impact our global estimates of *Zostera* seagrass distribution since *Z. japonica* is invasive and spreading rapidly throughout North America (Shafer, Kaldy & Gaeckle, 2014), and *Z. capensis* is identified as experiencing extensive losses (Mvungi & Pillay, 2019). Given the limited number of articles our review encountered for these species, data and research on the extent of these species may be lacking or inaccurate.

### Geographical hotspots of *Zostera* seagrass research exist within the review, and *via* search databases

Having mapped full-text article locations, we saw trends in disproportionate numbers of articles included in our review occurring at different geographical locations. The USA, Australia, and certain European countries were considerable research hotspots for *Zostera* articles identified by our review. In contrast, many other countries only recorded a single article, such as Chile and Turkey. Thus, we highlight countries and locations that had low articles included in our review and discuss a few reasons why this may be the case. These low article numbers from our review may not be an indication of low research effort at these locations, but rather (1) Web of Science may not index journals that academics from certain countries preferentially publish in (Mongeon & Paul-Hus, 2016); (2) there may be multiple species of seagrass in certain countries, and research effort may be directed towards species in other genera not included in our review (Bujang, Zakaria & Arshad, 2006); (3) there may be few occurrences of *Zostera* seagrass in these places due to low habitat suitability (Blanco-Murillo et al., 2023); and/or (4) some places may lack data or resources to conduct research on *Zostera* stressors (Unsworth, Nordlund & Cullen-Unsworth, 2019). While our review cannot pinpoint the exact reasoning for each of these locations’ low article numbers, we emphasize that biased indexing of certain journals could provide context for a lack of data centralization for *Zostera* seagrass research. Nevertheless, addressing these knowledge and centralization gaps is critical, and prioritizing increased monitoring and data collection in regions that have not had the resources to do so is essential to ensure a more equitable and comprehensive understanding of *Zostera* ecosystems worldwide.

### Certain stressors are studied in great depth, but the *Zostera* genus would benefit from a better understanding of all stressors, their relationships to one another, and species-specificity

We found temperature, light, and nutrient stressors were studied most frequently, both individually and with other frequently studied stressors. These patterns persist in our umbrella analysis of stressors, making stressors categorized as pollution and climate change a popular area of study genus-wide. In looking at our stressor definitions and umbrella categories, it is important to note that many of these stressors are largely caused by anthropogenic sources, echoing findings from Tang & Hadibarata (2022). The three top stressors from our review are major influencers of *Zostera* seagrass growth, affecting numerous biological processes within the marine macrophyte (Lee, Park & Kim, 2007). *Zostera*, specifically, has one of the highest known light requirements of marine primary producers; therefore, studying this stressor has enhanced understanding of its influence on growth dynamics and losses (Bertelli & Unsworth, 2018). Temperature, light, and nutrients influence seagrass growth (Lee, Park & Kim, 2007; Kaldy, 2014); thus, studies focus on these stressors to understand the distribution and seasonal changes in seagrasses (Hirst et al., 2017). However, because these stressors have also been closely associated with eutrophication, macroalgal presence, and light limitation (Han et al., 2016; Mvungi & Pillay, 2019), research often focuses on these factors to better understand the anthropogenic impacts driving seagrass declines (Wong, Griffiths & Vercaemer, 2020). Given the importance of these three stressors as baseline population controls and as stressors with negative impacts when exacerbated by anthropogenic influences, it is understandable why they are the most commonly studied stressors in the literature.

Our study made significant progress in summarizing the current state of knowledge regarding stressors and seagrasses in the *Zostera* genus, particularly with how they relate to multiple aspects of research studies. In our analysis of stressors by the species they were studied with, nutrients were most frequently studied with six of the top eight *Zostera* species, demonstrating an overall trend in the study of nutrients with many species of *Zostera.* The frequent study of nutrients with most species in this genus may suggest a consensus within the field regarding the impacts of nutrients as a stressor, both for seagrasses specifically (Vieira et al., 2020) and for global oceans more broadly (Halpern et al., 2012). Thus, the frequent study of nutrients is warranted and should continue, not only because of its role in ocean health, but also because management practices aiming to reduce nutrient load have shown success and greater feasibility in comparison to other stressors (Orth et al., 2006; Duarte & Krause-Jensen, 2018). Although the research emphasizes that managing other stressors can play a role in seagrass ecosystem recovery, this knowledge will be useful to managers and policymakers in situations where they need to implement regulations prior to primary research being conducted for their specific species and/or location. We indicate that geographic and species-specific research will be most helpful for informing management strategies targeted towards specific species or regions; however, utilizing the knowledge and management options available may be a practical strategy for temporarily filling knowledge gaps.

Recent studies suggest stressors often co-occur rather than existing as single stressors, and that multiple stressors can have synergistic, antagonistic, additive, or unknown cumulative impacts in seagrass ecosystems (Griffen et al., 2016). Stockbridge, Jones & Gillanders (2020) showed a general lack of centralized data availability on co-occurring stressors in seagrass, and a heightened need for conservation actions to take multiple stressors and more frequently occurring stressors into account for effective results. From our own review, we did find increases in the number of studies measuring more than one stressor over publication time; the number of studies measuring more than one stressor exceeded those of single stressor studies in almost every year after 2000. This is a very positive finding for the *Zostera* genus, demonstrating that researchers have likely reached a consensus about the importance of multiple stressors. While we did not quantify the interactions between stressors, like Stockbridge, Jones & Gillanders (2020), our findings provide a unique perspective on *Zostera* genus-wide trends of study for numerous broadly defined stressors, complementing the results of Stockbridge, Jones & Gillanders (2020). Together, these papers can inform future research efforts and conservation practices for the *Zostera* genus.

Often-studied stressors will be important given the greater data availability and overlap of these stressors. For example, sediment and light are frequently studied in the same papers (Richter, Perdue & Patterson, 1995; Palinkas & Koch, 2012) and are often associated when viewing turbidity as an overarching threat to seagrasses. Many of these factors do not occur alone; however, it is important to note the greater data availability for some may bias our overall understanding of *Zostera* seagrasses. Therefore, the stressors we most frequently recorded can help guide future research with the less-studied stressors, which together, may offer better data and insights.

Despite low article numbers, it is hard to discount certain stressors, such as pathogens, including seagrass wasting disease (*Labyrinthula zosterae*) (Graham et al., 2021). Seagrass wasting disease was first observed in the Atlantic population of *Z. marina* in the 1930’s (Muehlstein, Porter & Short, 1988), causing critical losses (90% of the Atlantic distribution); however, we did not record an article for “pathogen” stressors until 1978 (Kirkman, 1978). Although we did see small increases in pathogen stressor articles over time, these numbers remained relatively low, and in general, this field is understudied with the full extent of pathogens and their impacts on seagrasses unknown (Sullivan et al., 2018). Given the severity of losses caused by wasting disease, further research can improve our knowledge of seagrass pathogen prevalence (Short, Ibelings & Hartog Den, 1988). We also did not find many articles studying the microbial dysbiosis as a stressor. This is concerning since clear impacts from the microbiome have been established for other organisms, including terrestrial plants and corals (Ugarelli et al., 2017). Although much remains unknown about the seagrass holobiont, some studies reported the microbiome’s ability to produce bio-available nutrients and act as a stressor *via* biofilm formation (Hurtado-McCormick et al., 2019; Tarquinio et al., 2019). The study of microbiomes in seagrass is a relatively new field and has necessitated technological advancement (Ugarelli et al., 2017). We did not record an article for “microbial dysbiosis” until 1996 and only saw small increases over time (Fig. S7). Finally, there is growing support for the idea that other stressors, including temperature, nutrients, and light (our top most-studied stressors), may exacerbate seagrass vulnerability to pathogens and infection, and in other scenarios, that the microbiome may be able to ameliorate some effects of stressors on seagrasses (Sullivan et al., 2018). Therefore, we emphasize that the stressors studied at lower frequency should be investigated further, and when possible, considered in conjunction with our top-most studied stressors.

### Limited communication and centralized knowledge may have contributed to concentrated areas of study

One of the main takeaways from our review is the lack of research and data cohesion that exists for seagrass in the *Zostera* genus. From contradictory taxonomic definitions to locations that lack stressor data, there is an imminent need for centralized agreement and resources to be used for defining *Zostera* seagrass species, centralizing current data, and to aid in informing research needs. Our review and synthesis generated a large sum of data that we feel will best serve the *Zostera* seagrass research and management fields as an open-access, data repository which will allow for continued use of the data. To begin, we registered and uploaded all our data onto Open Science Framework (Foster & Deardorff, 2017, http://www.osf.io/) for public access. Our code and workflows are also included on the Open Science Framework project page for method reproducibility and guidelines for new systematic reviews. Second, we included a link on OSF to our interactive systematic review map, which can be utilized by future studies to explore region-specific papers or global trends for stressors, study designs, species, and response variables. These two tools are first steps towards centralized agreement *via* a foundation of knowledge starting with our review of the *Zostera* genus but can be expanded to include other seagrass genera, and literature searches of different databases. Our goal is to have this database be scientifically resourceful and publicly informative to enhance *Zostera* seagrass management and future research initiatives. In our results and discussion, we show a few scenarios where this may be useful, including reference of response variables in study design, and to better inform species distribution of this genus.

To our knowledge, few global, *Zostera* seagrass-specific databases exist to date, with very few databases in general featuring ample peer-reviewed current information (but see Edwards, 2004) and ZEN (*Zostera* Experimental Network, http://www.zenscience.org). Both of the aforementioned sources made great efforts to focus on all plants, or a specific species of *Zostera*, but no such database, even our own, meets the needs we uncover here. Global, centralized knowledge of *Zostera* seagrass stressors is needed imminently as seagrasses face certain common challenges, but also to aid in targeted efforts for unique threats faced by specific species that will require local intervention to combat range shifts, invasions, and destructive land use practices (Unsworth, Nordlund & Cullen-Unsworth, 2019). From the knowledge gaps identified with our review, we recommend the following essential information for a *Zostera* database to feature: best practices for standardized response variable measurements, distribution data, study designs for assessing multiple stressors, and novel methods that may be implemented to further our understanding of *Zostera* species.

## Conclusion

Our findings from this systematic map have collated the tremendous body of work that currently focuses on the *Zostera* genus, while also highlighting concentrated areas of research and persistent knowledge gaps. We emphasize the need for greater research and conservation actions focused on *Zostera* seagrasses, particularly for species and stressors that had lower article numbers in our review. Research article numbers for seagrasses overall have been historically low in comparison to those of other coastal ecosystems, with 60% of all published research focused on coral *versus* the 11–14% focused on seagrass (Duarte et al., 2008). While these disparities have previously existed, we are hopeful for the future of *Zostera* seagrass research, given the consistently increasing number of articles with each publication year that we summarize with our review. Furthermore, hallmarks of our review’s findings point to patterns of diversifying study designs for a greater understanding of this genus, studies’ frequent use of similar response variables for comparable data, possible consensus about top stressors of concern for this genus, and the recognized need for consideration of multiple stressors. All these findings lead to a positive outlook on the direction that *Zostera* research is headed. However, it is still important to note that specific species, geographical, and stressor biases were identified in this review, which was compounded by a general lack of centralization of *Zostera* seagrass research. We underscore these important knowledge gaps to help direct priorities for *Zostera* conservation, research, and management so that we can move forward in collaborating and researching avenues to slow losses of current seagrass extent and create an inclusive understanding of *Zostera* seagrass ecosystems. Future, inclusive understandings should aim to encapsulate consultation and co-design of studies and management with Indigenous Peoples and local communities threaded together with expert knowledge, increasing equity of research across *Zostera* species, and understanding which stressors may be threats to the overall genus, as well as stressors that have gone understudied, or species-specific. Findings from this review have been transformed into an open-access, interactive map of studies, which is intended to facilitate research centralization for *Zostera* species and stressors in addition to allowing for public use of our data and findings. The benefits provided by seagrass meadows, including carbon sequestration, essential fisheries habitat, and water quality enhancement, make them some of the world’s most valuable coastal ecosystems, and necessary conservation targets (Barbier et al., 2011). Therefore, it will be vital to build upon the impressive foundation of studies summarized in this review by incorporating diverse and novel methodologies, standardizing response variables, as well as undertaking equitable research across species, stressors, and regions for the *Zostera* genus.

##  Supplemental Information

10.7717/peerj.19209/supp-1 Supplemental Information 1 PRISMA abstract checklist

10.7717/peerj.19209/supp-2 Supplemental Information 2 PRISMA checklist

10.7717/peerj.19209/supp-3 Supplemental Information 3 Outline of all supplemental figures, tables, protocols, and data

10.7717/peerj.19209/supp-4 Supplemental Information 4 Review code/methodology for title and abstract screening

10.7717/peerj.19209/supp-5 Supplemental Information 5 Protocol for full article screening and extraction

10.7717/peerj.19209/supp-6 Supplemental Information 6 Protocol for full article screening and extraction

10.7717/peerj.19209/supp-7 Supplemental Information 7 Example full-text extraction form

10.7717/peerj.19209/supp-8 Supplemental Information 8 Study type and study design descriptions

10.7717/peerj.19209/supp-9 Supplemental Information 9 Descriptions and examples of stressors to seagrasses, umbrella categories

10.7717/peerj.19209/supp-10 Supplemental Information 10 Response variable descriptions and examples

10.7717/peerj.19209/supp-11 Supplemental Information 11 Articles excluded at title and abstract screening phase

10.7717/peerj.19209/supp-12 Supplemental Information 12 Articles excluded during full-text screening

10.7717/peerj.19209/supp-13 Supplemental Information 13 Title and abstract screening decision tree All article screeners were trained using this workflow, inclusion or exclusion of each article required two screeners to use this tree and arrive at the same conclusion for exclusion or inclusion of an article.

10.7717/peerj.19209/supp-14 Supplemental Information 14 Full-text screening decision tree All article screeners were trained using this workflow in addition to 10 practice articles; inclusion or exclusion of each article required a screener to use this tree, make a decision to include or exclude the article, then extract data from the article if an include decision was made.

10.7717/peerj.19209/supp-15 Supplemental Information 15 Full-text screening extraction fields All extraction fields for data extraction during full-text article review. Fields were contained in the form of an excel sheet, where reviewers had drop-down options for selection, eliminating variation between reviewers. Supplemental information S4 also contains an example of the data extraction sheet.

10.7717/peerj.19209/supp-16 Supplemental Information 16 Frequency of study type and study design over all publication years for full text articles Each of the five study type and design combinations are displayed with data points for the publication years 1938–2021. Most 2021 publications were pre-prints at the time of literature search, and article numbers are only representative up until the date of the search execution, meaning the 2020 and 2021 numbers may not be completely represented by our review.

10.7717/peerj.19209/supp-17 Supplemental Information 17 Number of articles per publication year for included full-text articles, and two other coastal ecosystems Number of articles per publication year for included full-text articles (*Zostera*-stressor), and a Web of Science search for: genus (*Zostera*), seagrass overall (Seagrass), and two other coastal ecosystems (Coral, Mangroves). Article numbers per publication year were obtained from a Web of Science search for each respective term. Most 2021 publications were pre-prints at the time of literature search, and article numbers are only representative up until the date of the search execution, meaning the 2020 and 2021 numbers may not be completely represented by our review.

10.7717/peerj.19209/supp-18 Supplemental Information 18 Number of stressors recorded for review articles, broken down by publication year (A) Publication years for articles that measured one stressor, compared to articles that measured more than one stressor. (B) Publication years for articles broken down by the exact number of stressors recorded (1, 2, 3, 4+). Most 2021 publications were pre-prints at the time of literature search, and article numbers are only representative up until the date of the search execution, meaning the 2020 and 2021 numbers may not be completely represented by our review.

10.7717/peerj.19209/supp-19 Supplemental Information 19 Number of articles by publication year for 23 individual stressors included in the review (1938-2021) Grid ordered by total articles for each individual stressor within the four umbrella categories, highest to lowest. Stressors colored by umbrella categories: pollution (orange), climate change (red), increased anthropogenic presence (pink), and intrinsic factors (blue).

10.7717/peerj.19209/supp-20 Supplemental Information 20 Response variables that were investigated by full text articles Individual type and counts of each response variable were sorted into categories of plant, community, and environment recorded in combination by each included article. Combinations included: Plant, Community, or Environment only; Plant & Community (PC); Plant & Environment (PE); Community & Environment (CE); or Plant, Community, & Environment (PCE).

10.7717/peerj.19209/supp-21 Supplemental Information 21 Interactive map

## Additional Information and Declarations

### Data Availability

The following information was supplied regarding data availability: The data is available at Open Science Framework: Lyford, HR., Guerrini Romano, E., Sura, SA., & Bittick, S. J. (2023, October 15). Understanding trends in seagrass research, stressors, and response variables: a global systematic review of the genus Zostera. Available at osf.io/3cjrf.
