# Supplementary material for: Understanding trends in Zostera research, stressors, and response variables: a global systematic review of the seagrass genus
Source: PeerJ. 2025 Apr 17;13:e19209. doi: 10.7717/peerj.19209 (PMC12009562; doi:10.7717/peerj.19209)
Supplement: Supplemental Information 3 [file peerj-13-19209-s003.docx]

**Supplemental Information 1.3, Supplement Outline**

1. Supplemental Information 1: PRISMA Checklists, Supplement Outline
   1. 1.1 PRISMA abstract checklist
   2. 1.2 PRISMA checklist
   3. 1.3 Supplemental Information Outline
2. Supplemental Information 2. Review code / methodology for title and abstract screening.

2.1 Upload Protocol

2.2 Title & Abstract Screening Protocol

2.3 Tagging Articles with Keywords

References

1. Supplemental Information 3. Protocol for full article screening and extraction

3.1 Full Text Screening protocol

3.2 Examples of Excluded Studies

3.3 Data Extraction Protocol

References

1. Supplemental Information 4. Full-Text data extraction forms, study type & study design descriptions

4.1 Completed Full-Text Extraction Form (Excel File)

4.2 Example Full-Text Extraction Form (Excel File)

4.3 Study Type and Study Design Descriptions

1. Supplemental Information 5: Supplemental Information 5. Descriptions and examples of stressors to seagrasses, umbrella categories.

5.1 All Stressor Descriptions

5.2 Description of Umbrella Categories of Stressors

References

1. Supplemental Information 6: Response Variables

6.1 Plant Response Variable Descriptions

6.2 Community Response Variable Descriptions

6.3 Environment Response Variable Descriptions

References

1. Supplemental Information 7: Records of Excluded Articles

7.1 Excel Sheet of Articles Excluded at Title and Abstract Screening Phase

7.2 Excel Sheet of Articles Excluded During Full-Text Screening

Note: All data, analysis, and code can be found on our open science framework page,

<https://osf.io/3cjrf/?view_only=f2502a2d54cf49349f3f16f7ad1495e5>
