# Supplementary material for: Understanding trends in Zostera research, stressors, and response variables: a global systematic review of the seagrass genus
Source: PeerJ. 2025 Apr 17;13:e19209. doi: 10.7717/peerj.19209 (PMC12009562; doi:10.7717/peerj.19209)
Supplement: Supplemental Information 4 [file peerj-13-19209-s004.docx]

# **Supplemental Information 2. Review code / methodology for title and abstract screening.**

**SI 2.1 Exporting Abstracts & Citations from Web of Science to Colandr:**

1. After executing the search, scroll to the bottom of the search page.
2. Select export to other file formats.
3. Export up to 500 articles at a time.
4. Select option where abstracts are included.
5. Export in File format as “other reference software.”
6. Make sure the file is exported as .RIS or .txt
7. If .txt is not working, import to Zotero and re-export to .RIS for Colandr
8. Import the .ris or .txt file to Colandr. Repeat for all articles.

**SI 2.2 Protocol for Screening Titles and Abstracts:**

1. Upon logging on to Colandr, select the appropriate review.
2. Enter the “review progress” page.


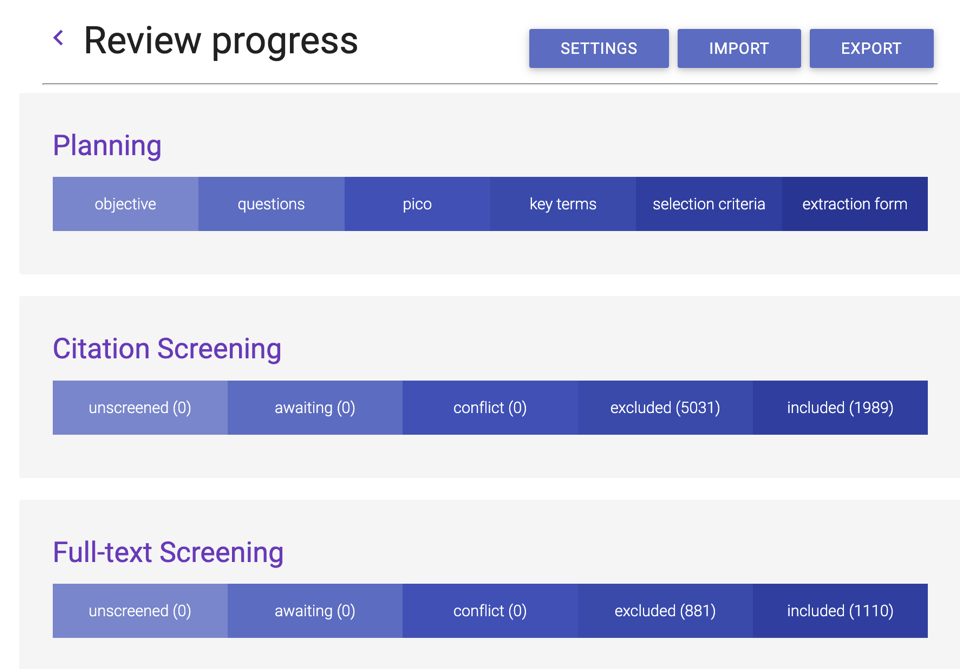


1. Select “unscreened articles” tab.


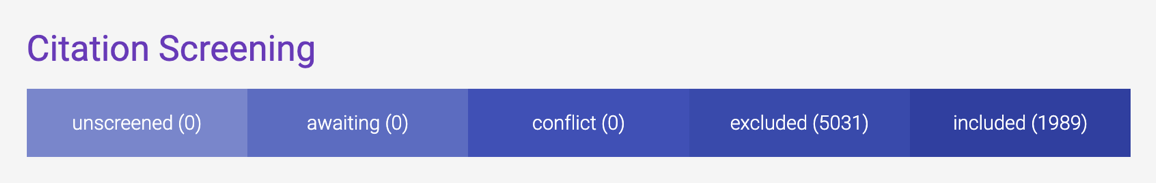


1. Select an article at the top of the unscreened articles page.
2. Read through the title and abstract of the article.


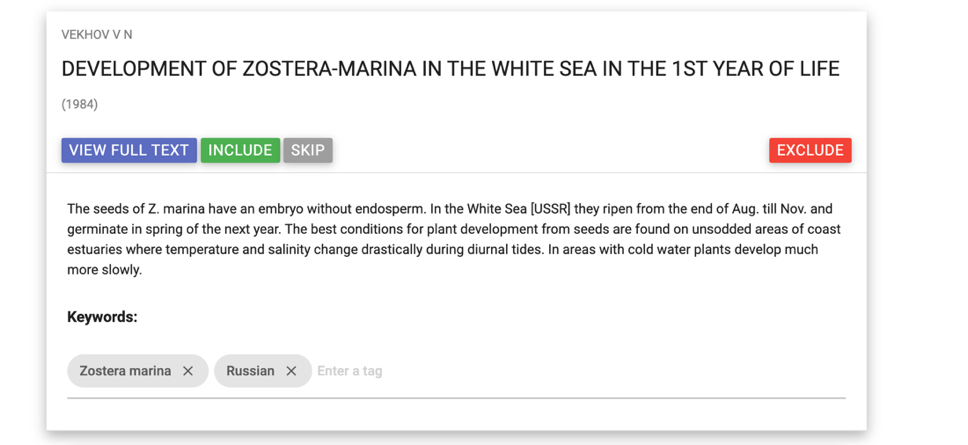


1. Use the title and abstract decision tree to determine the article’s relevance
2. If the article fits the decision tree’s include criteria
3. If it is decided that the article is to be included, enter in keywords for the species (or multiple), stressor(s), and any article type or language aside from English. (All keywords listed below)
4. After keywords have been entered, select “include”
5. If the article is to be excluded, select exclude-a drop down menu will appear


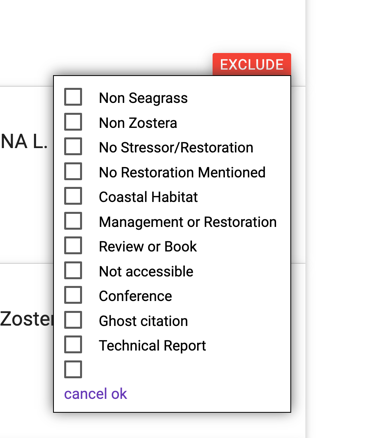


1. Use the options that correlate to the decision tree to select why the article was excluded (only select one)
2. Select “ok”
3. If unsure, click “skip” or consult the review developers.

**SI 2.3 Keyword Tags to be used for Colandr:**

Stressors:

- Light
- Temperature
- Sediment
- Salinity
- Nutrients
- Acidification
- Carbon
- Aquaculture Impacts
- Hydrodynamics
- Oxygen
- Herbivory
- Pathogens
- Habitat Fragmentation
- Invasion
- Drought
- Epiphytes
- Invertebrates
- Herbicides
- Genomics
- Toxins
- Seagrass competition
- Microbiome
- Anthropogenic Use
- Stressor (other not listed)

Species

- *Zostera angustifolia*
- *Zostera asiatica*
- *Zostera* c*aespitosa*
- *Zostera capensis*
- *Zostera capricornii*
- *Zostera capricorni*
- *Zostera caulescens*
- *Zostera chilensis*
- *Zostera japonica*
- *Zostera kiewiensis*
- *Zostera mucronata*
- *Zostera marina*
- *Zostera mulleri*
- *Zostera muelleri*
- *Zostera nigricaulis*
- *Zostera noltii*
- *Zostera nolti*
- *Zostera novazelandica*
- *Zostera pacifica*
- *Zostera polychlamys*
- *Zostera tasmanica*

Article Characteristics of Interest:

- Technical Report
- Review
- Model
- Ghost Citation
- Conference

Language

- If an article is in a language other than English, please enter the language into the keywords.

Tagging an article:

1. Go to article (unscreened) list
2. Click on desired article title
3. Abstract will appear below the title, scroll to below the abstract
4. There will be a line that says keywords
5. Type in desired tag(s) and hit enter
6. Tag(s) will be saved automatically

**References:**

https://www.colandrapp.com

Cheng, SH., Augustin, C., Bethel, A., Gill, D., Anzaroot, S., Brun, J., DeWilde, B., Minnich, R., Garside, R., Masuda, Y., Miller, DC., Wilkie, D., Wongbusarakum, S. and McKinnon, MC. (2018), Using machine learning to advance synthesis and use of conservation and environmental evidence. *Conservation Biology*, 32: 762-764. doi:10.1111/cobi.1311

Certain data included herein are derived from Clarivate Web of Science. © Copyright Clarivate 2022. All rights reserved.
