# Supplementary material for: Understanding trends in Zostera research, stressors, and response variables: a global systematic review of the seagrass genus
Source: PeerJ. 2025 Apr 17;13:e19209. doi: 10.7717/peerj.19209 (PMC12009562; doi:10.7717/peerj.19209)
Supplement: Supplemental Information 5 [file peerj-13-19209-s005.docx]

**Supplemental Information 3. Protocol for full article screening and extraction.**

**SI 3.1: Protocol for full article screening**

1. Login to colandrapp.com and click on “Unscreened” under Full Text Screening


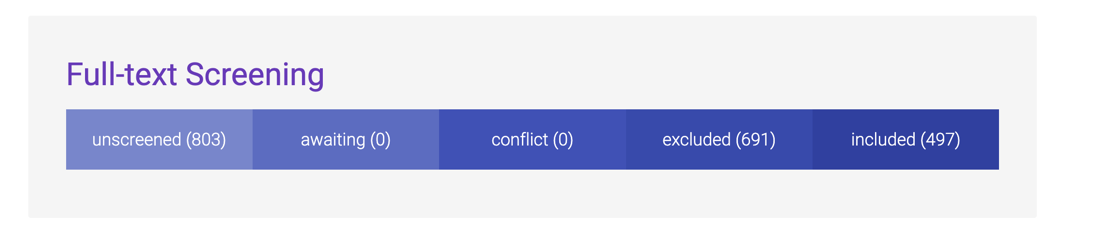


2. Select an article and click “View Full Text” to read it.


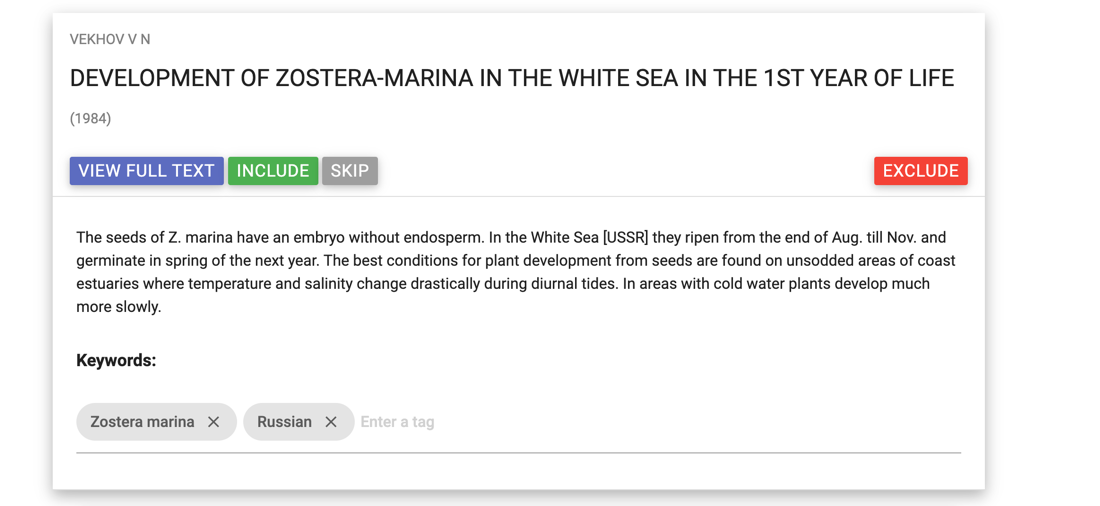


3. Use the decision tree below to decide whether to include (and extract data) or exclude the

article.


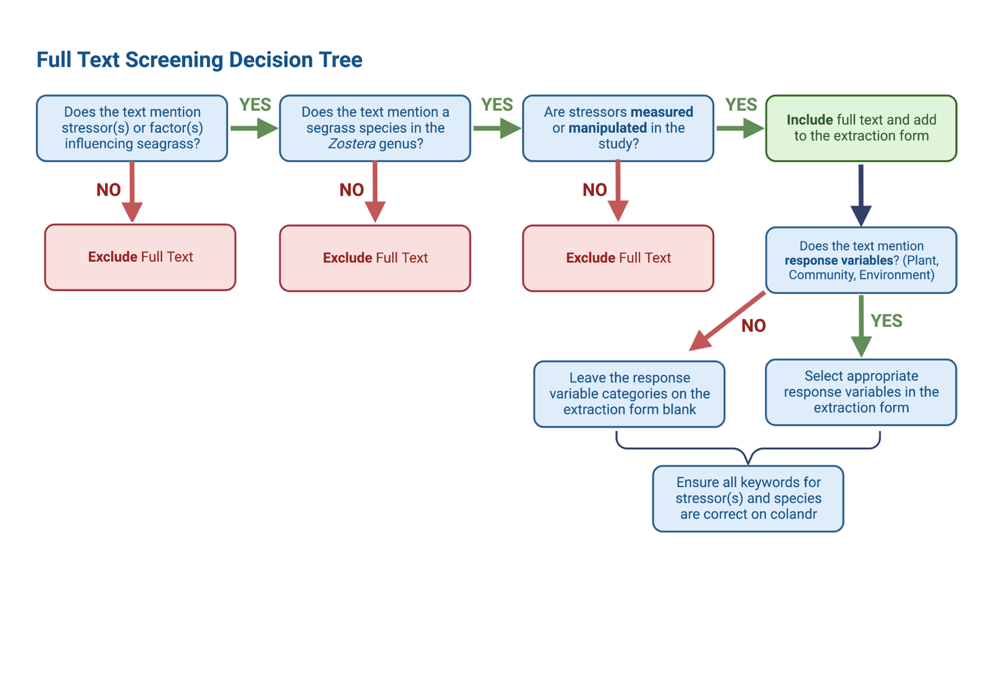


**4.** If the article should be excluded: click exclude and select the appropriate reason. For example, if the article is not about any species in the genus *Zostera*, but involves restoration or management/conservation of a different genus of seagrass, click “Exclude” and select the “Non Zostera” and “Management or Restoration” boxes as the reason. If you are not sure of the exclusion reason, or if an article should be excluded consult the study leads. If the article is excluded, end here with this article and go back to step 1.


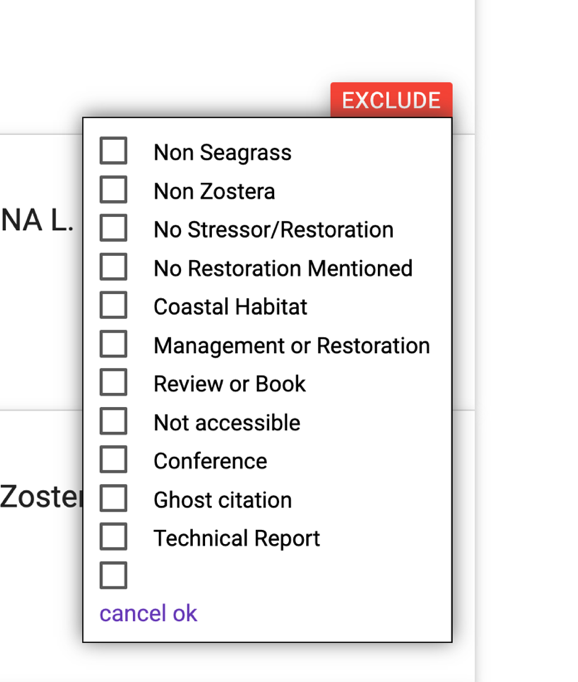


If the article should be included, see the instructions below.

**SI 3.2: Examples of articles that may seem like they might be included, but should be excluded**

- Nutrient cycling in the water column of a subtropical seagrass meadow Ziegler, S., & Benner, R. (1999).
  - Explanation: While this article does mention nutrients (one of our stressors), and focuses on seagrass, **the seagrass species** is the reason for exclusion. This article looked at *Thalassia testudinium*, which is not a species in *Zostera* genus.
  - Exclusion Reason: Non-Zostera
- Conservation genetics of Australasian sailfin lizards: Flagship species threatened by coastal development and insufficient protected area coverage. Siler, Lira-Noriega, & Brown (2014).
  - Explanation: This article has a brief mention of seagrass, but focuses on lizards. Also, there were no stressors to seagrass mentioned, and no genus for the seagrass.
  - Exclusion Reason: Non-Seagrass
- Isolation and characterization of microsatellite markers for Zostera marina and their cross-species amplification in Zostera caespitosa. Peng et al. (2012).
  - Explanation: This article mentions seagrass, and both seagrasses mentioned are in the *Zostera* genus. However, there are no stressors studied in this paper, it is just looking at microsatellite markers. Therefore, it should be excluded.
  - Exclusion Reason: No Stressor

**SI 3.3: Steps for extracting data from included articles**

1. If you have made it to this point, you should be at the green box in the upper right (don’t click Include yet!)
2. Add the article to your individual extraction sheet under the “Extraction Form” tab. The Metadata tab can help you make sure to input the required data in the appropriate format but the instructions are also here.
3. The following categories should be **typed in** according to the format described by the metadata form:

- Title
- Author(s)
- Publication Year
- Date Assessed
- Person Extracting the Data
- Name of Location
- Location-GPS coordinates
- Latitude Longitude

1. **IMPORTANT:** Please make sure latitude and longitude columns are in the correct format. You can find the latitude and longitude as real numbers by searching the location in Google Maps or using this conversion calculator from GPS units to real +/- numbers: <https://www.pgc.umn.edu/apps/convert/> . The northern hemisphere is positive, and the southern hemisphere is negative latitude; the eastern hemisphere is positive and the western hemisphere is a negative latitude. **Double check this for accuracy.**

**If your article has more than one site, the end of the sheet (column AF to BG) has spots for adding these sites, up to 8 locations. Make sure to insert the appropriate values to the latitude and longitude columns.


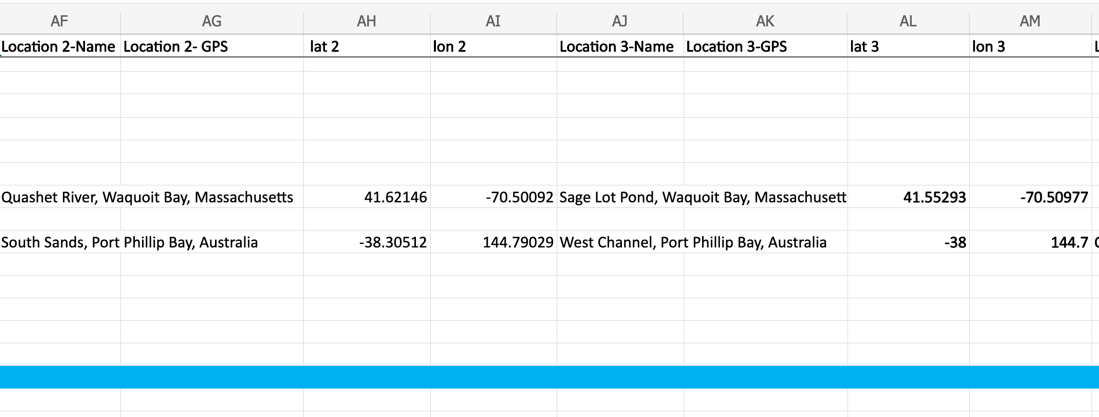


1. The following categories should be entered **using the drop-down list** on the extraction form. The photo below shows an example of how the drop down menu should appear for the following categories on the extraction form.

- Study Design
- Study Type
- *Zostera* Species
- Stressor(s)
- Category and Combination of Plant, Community, or Environmental Response Variables.
- Response Variables (Plant)
- Response Variables (Community)
- Response Variables (Environment)


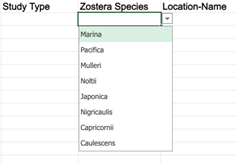


1. The Categories Study Design, Study Type, and *Zostera* species **should all be filled in** with a drop-down answer. If the dropdown feature is not working on your sheet for these columns—please contact study leads.
2. The stressor and response variable categories, (categories with 4 columns) do not need to be completely filled in unless there are 4 stressors, 4 Plant Response Variables, etc.
3. If there is a species, stressor, or response that is not included in the dropdown choices or surpasses the number allowed, let the study leads know and also add it to the addition columns in your sheet.


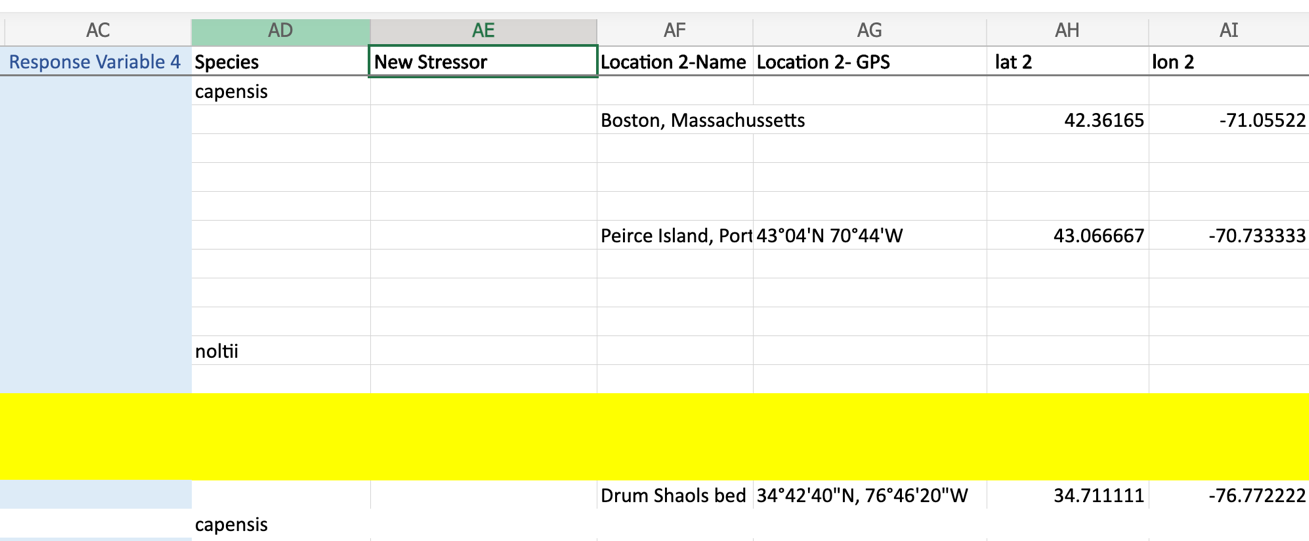


1. Review the keywords on the article in Colandr. Stressors and species should be included here. Ensure the species and stressors match the inputs in your extraction sheet. Do not include keywords that do not match your extraction sheet, do not include response variables.


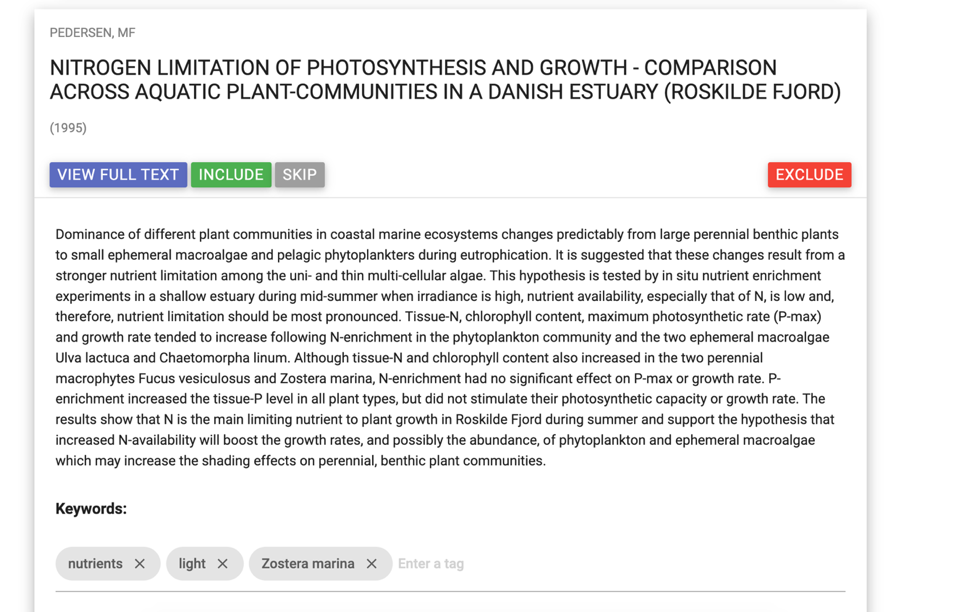


1. Once the keywords are appropriately added, click include. The article will now move from the “Unscreened” category to “Included”.
2. Ensure everything is consistent and appropriate in your extraction sheet, and on Colandr. The study leads will add your sheet rows to the master datasheet, therefore the columns must line up.
3. Once finished excluding/including an article and extracting data, return to Step 1.

**References**

<https://www.colandrapp.com>

Cheng, SH., Augustin, C., Bethel, A., Gill, D., Anzaroot, S., Brun, J., DeWilde, B., Minnich, R., Garside, R., Masuda, Y., Miller, DC., Wilkie, D., Wongbusarakum, S. and McKinnon, MC. (2018), Using machine learning to advance synthesis and use of conservation and environmental evidence. *Conservation Biology*, 32: 762-764. doi:10.1111/cobi.1311

Google Earth, <https://earth.google.com/web/>, version 9.194.0.0. Data Retrieved June 2020-August 2023.

Peng, J., Zhang, L., Jiang, X., Cui, C., Wu, R., & Zhao, J. (2012). Isolation and characterization of microsatellite markers for Zostera marina and their cross-species amplification in Zostera caespitosa. *Conservation Genetics Resources*, *4*, 455-458.

Siler, C. D., Lira-Noriega, A., & Brown, R. M. (2014). Conservation genetics of Australasian sailfin lizards: Flagship species threatened by coastal development and insufficient protected area coverage. Biological Conservation, 169, 100-108.

Certain data included herein are derived from Clarivate Web of Science. © Copyright Clarivate 2022. All rights reserved.

Ziegler, S., & Benner, R. (1999). Nutrient cycling in the water column of a subtropical seagrass meadow. *Marine Ecology Progress Series*, *188*, 51-62.
