# Supplementary material for: Understanding trends in Zostera research, stressors, and response variables: a global systematic review of the seagrass genus
Source: PeerJ. 2025 Apr 17;13:e19209. doi: 10.7717/peerj.19209 (PMC12009562; doi:10.7717/peerj.19209)
Supplement: Supplemental Information 8 [file peerj-13-19209-s008.docx]

**Supplemental Information S4**

**SI 4.3 Study Type and Study Design Descriptions**

Study type and study design of the research were recorded during full-text screening for us to draw conclusions on how most studies were conducted. Articles included empirical studies, review articles, technical reports, and model studies if original data were presented. We established types of studies as model, field, and mesocosm studies, while we divided design between survey and manipulative studies. We defined model studies as those using mathematical, spatial tools, or a combination of the two to expand upon empirical data, leading to a conclusion regarding stressor effects on a *Zostera* species. We defined field studies as those conducting the study in the natural environment (*in situ*) where there is little control over naturally occurring variables. We defined mesocosm studies as study types conducted in an artificial environment, such as a lab or outdoor tanks, where one or more variable, or stressor could be controlled to a greater degree. For study design, manipulative studies changed independent variables via addition or removal (perturbation), while surveys recorded data without changing any independent variables (observational). Field and mesocosm studies could be recorded in combination with either survey or manipulative designs. If a study reported on more than one type or design conducted, we followed a hierarchy to determine the single study type and study design that were recorded. For study types that reported on both mesocosm and field studies, mesocosm was recorded. For study designs that reported on both manipulative and survey studies, manipulative was recorded for that article. Therefore, each study type and study design field were associated once with each article.  The combination of mesocosm-survey study type-design was only found for 10 research articles. Although this combination may be counterintuitive, these studies were characterized this way because they aimed to measure in-situ conditions in a mesocosm environment. For example, Nielson et al (2002) had this experimental design in order to measure sulfate reduction/nitrogen fixation rates in mesocosms containing collected seagrass and sediment.

Nielsen, L. B., Finster, K., Welsh, D. T., Donelly, A., Herbert, R. A., De Wit, R., & Lomstein, B. A. (2001). Sulphate reduction and nitrogen fixation rates associated with roots, rhizomes and sediments from Zostera noltii and Spartina maritima meadows. *Environmental Microbiology*, 3(1), 63-71.
