# Supplementary material for: Understanding trends in Zostera research, stressors, and response variables: a global systematic review of the seagrass genus
Source: PeerJ. 2025 Apr 17;13:e19209. doi: 10.7717/peerj.19209 (PMC12009562; doi:10.7717/peerj.19209)
Supplement: Supplemental Information 9 [file peerj-13-19209-s009.docx]

**Supplemental Information 5. Descriptions and examples of stressors to seagrasses, umbrella categories.**

**5.1 Stressor Descriptions**

Light

- This stressor has multiple impacts and could be light affecting seagrass as a limiting factor, or in excess. This is seen as an abiotic impact in studies looking at seasonal light availability, in the field or simulated (usually associated with temperature (Verhagen & Nienhuis, 1983 and Conover, 1958). Light was also commonly mentioned in studies as limited by a nutrient (commonly associated stressor) influx which caused a macroalgae bloom resulting in light limitation (van Lent et al., 1995).

Temperature

- This is seen as an abiotic impact in studies looking at seasonal temperature fluxes, in the field or simulated (usually associated with light) (Sand-Jensen, 1975). This could also be associated with rising water temperatures discussed in relation to climate warming (Johnson et al., 2021).

Sediment

- Sediment can be associated with seagrass in contributing to water turbidity, studied for its characteristics of accumulation in meadows, or development of anoxic or toxic patches (Terrados et al., 1999). Sediment was often tagged as a stressor in addition to light due to it’s light limitation capability (McGlathery *et al*., 2013).

Salinity

- Extreme salinities both low and high were studied in field and mesocosm studies 
  looking at how the stressor limited distribution and growth. Could be from 
  greater evaporation (high salinity) or looking at Zostera growth in brackish 
  waters. (Sola et al., 2020). This is also often associated with hydrodynamics and temperature (Kotta *et al.,* 2014).

Nutrients

- Nutrients were measured as an anthropogenic input such as runoff or fertilizer, causing macroalgal blooms (Harlin & Thorne-Miller, 1981), or seasonally looking at nitrogen as a limiting nutrient for plant growth (Zimmerman, Smith, & Alberte, 1987). Nutrients can also be a stressor in tissue and plant breakdown (Pellikaan & Nienhuis, 1988).

Acidification

- (Ocean) acidification is the decrease in pH mainly due to increases in carbon dioxide inputs to the system. The acidification could be studied as a stressor to the community surrounding seagrass, or as impacting the productivity of seagrass (Miller, Yang, & Love, 2018 and Collier et al., 2018). Acidification was a stressor often recorded in conjunction with carbon and hydrodynamics (Egea et al., 2018).

Carbon

- Carbon was studied in two main regards as a stressor, one as a limitation to seagrass growth, and another within the realm of carbon dioxide as a contributor to ocean acidification (Palacios & Zimmerman, 2007 and Jiang et al., 2017). Carbon was also often studied with an interest in seagrass blue carbon stocks, related to the study of limiting growth factors (Ferguson et al., 2017).

Aquaculture Impacts

- Studied as a stressor of seagrass related to the farming of fish, shellfish, and other aquatic organisms for commercial use. The stressor often stems from the releases of nutrients due to this activity (De Casabianca, Laugier, & Marinho-Soriano, 1997)

Hydrodynamics

- Hydrodynamic stressors of seagrass include wave action, storms, and water flow impacts on seagrass (Paulo et al., 2019). This can also be related to hydrodynamics and sediment, which can alter light and water turbidity (Hansen & Reidenbach, 2013).

Oxygen

- Oxygen stressors include hypoxic environments that may alter seagrass growth or survival (Lemley, Snow, & Human, 2014). Also studied could be sulfide toxins that reduce oxygen in seagrass tissues, studied in relation to metabolism (Pedersen, Binzer, & Borum, 2004).

Herbivory

- Herbivory as a stressor can be related to herbivores eating epiphytes off of seagrass leaves, altering growth (Hootsmans & Vermaat, 1985). Or, herbivory can be a stressor where herbivores are consuming seagrass (Lewis & Boyer, 2014).

Pathogens

- Pathogens can be found as stressors typically having to do with seagrass wasting disease, a protist pathogen, *Labyrinthula zosterae* (Brakel et al., 2019). This stressor is known to historically contribute to large seagrass die-offs and lower distribution. It is important to distinguish between pathogens and the microbiome, although they may be associated and both be selected. The seagrass wasting disease is most often associated with *Zostera marin*a (McKone & Tanner, 2009).

Habitat Fragmentation

Habitat fragmentation is often a stressor that interrupts connectivity between seagrass meadows, and destroys parts of the meadow, decreasing functionality (Beem & Short, 2009). This also includes disturbance/recovery experiments. Sometimes habitat fragmentation can be a study of function in bare vs. seagrass vegetated sites or as the removal of seagrass in a manipulative experiment (Aoki & McGlathery 2018). Furthermore, edge effects may play a key role in this dynamic, since the proportion of edge habitat is increased (Carroll et al., 2018)

Invasion

- Invasion stressors result from invasive species impacts to seagrass meadows, this can be in the form of another seagrass species, such as *Zostera japonica* (Hahn, 2003). Invasion can also be colonization by a non-invasive species such as tunicates (Carman et al., 2019).

Drought

- Drought as a stressor is often studied in relation to hydrodynamics, both can reduce freshwater or nutrient inputs to the seagrass meadows (Hirst et al., 2016).

Epiphytes

- Stressors that can grow on top of seagrass leaves and limit light penetration, and in turn growth (Hootsmans & Vermaat, 1985). Epiphytes can be algae, crustaceans, bacteria and can also be altered and affect seagrass via grazers (Jaschinski & Sommer, 2010).

Invertebrates

- Invertebrates often reside in seagrass ecosystems and include organisms such as crustaceans, mollusks, and echinoderms. They can be defined as the impact of one invertebrate on seagrass as a plant or community; or defined as the impact of abundance/diversity on a process.  Common incidences of invertebrates as stressors seagrass include consumption of seagrass, harvesting of invertebrates, as epiphytes, or as invasive species, altering the seagrass ecosystem. (Borowitzka, Lavery, & Keulen, 2006, Lewis & Anderson, 2012, Nakaoka, 2005, Liu, Pearce, & Dovey, 2015, and Ruesink et al., 2006). With this, sometimes herbivory, predation, and invasion will also be selected with the invertebrate stressor because the invertebrate fits into one of these categories as well (Amundrud et al., 2015 and Momota & Nakaoka, 2017)

Herbicides

- Herbicides are toxic to plants, commonly used in agriculture, and make up the majority of pesticides utilized worldwide (Dayan, 2019). Herbicides can be stressors to seagrass when runoff carries herbicides into meadows, inhibiting photosynthesis, and in large concentrations can cause plant mortality (McMahon et al., 2005). Hydrodynamics can often be studied in conjunction with herbicides as a stressor.

Genetic Diversity Loss

- Genetic diversity can be a stressor in that the diversity of a meadow or population determine resilience, or greater restoration success (Procaccini, Olsen, & Reusch, 2007).  Also, certain traits in the genetic makeup of seagrasses may be indicative of some species ability to tolerate/not tolerate certain environmental conditions (Pazzaglia et al., 2021).  Loss of diversity can have synergistic effects with other stressors, as well as decreasing reproductive success, and increased risk of extinction (Williams, 2001 and Elso, Manent., & Robaina, 2018).

Toxins

- Often found in sediments, or released into the water column, toxins can cause seagrass mortality and limited growth. Toxins to seagrass are often metals, or sulfides and can be pollutants, including runoff (Lee & Dunton, 2000 and Mylona et al., 2020).

Seagrass competition

- Seagrass competition can be intra- or inter-specific, as the result of either high-density meadows limiting production, competition with another species of seagrass for resources, or an invasive species competing with a native seagrass meadow (Rose & Dawes, 1999, Ruesink et al., 2010, and Sugimoto et al., 2017).

Microbial Dysbiosis

- The microbiome can be looked at in the scope of seagrass within the plant was well as directly surrounding the plant. Fungi, microalgae, and bacteria can associate with seagrasses and make up the plant microbiome: they can form mutualistic relationships that protect the plant from environmental extremes, or increase productivity (Hurtado-McCormick, 2019). Microbes can also be pathogenic to seagrasses or promote a worsening of stressor effects on the seagrass plants when the community is altered (Inaba et al., 2017 and Seymour et al., 2018).

Anthropogenic Use

- Anthropogenic uses of seagrass can include fishing, tourism, boating, oil spills, dredging, or building, all spanning of human uses acting directly or indirectly on seagrass meadows. Fishing can reduce fish diversity, while boat blades can scour seagrass blades, tourism may result in unintended consequences of human presence/usage in seagrass meadows, and oil spills can cause seagrass mortality (Icarella et al., 2018, Turschwell et al., 2021).

**5.2 Umbrella categories of stressors.**

Note, these stressors may be able to fit more than one category, however were classified in the category which the authors agreed was the best fit. Stressors can occur in isolation, or together. Umbrella categories were formed based off of categories from Kappel (2005)’s list of marine threats.

Pollution

- Description: abiotic stressors related to contamination, agricultural runoff, debris, often resulting in poor water quality.
  - Nutrients
  - Sediment
  - Toxins
  - Herbicides

Climate Change

- Description: Stressors that are either direct or indirect effects of global climate change (anthropogenic caused).
  - Light
  - Temperature
  - Hydrodynamics
  - Salinity
  - Oxygen
  - Invasion
  - Acidification
  - Drought

Increased Anthropogenic Presence

- Description: stressors to seagrasses derived from increases in human activity. These can typically occur from direct actions harming seagrasses.
  - Aquaculture Impacts
  - Habitat Fragmentation
  - Anthropogenic Use

Intrinsic Factors

- Description: stressors to seagrasses that may occur “naturally,” or exist as typical ecosystem processes but may result in seagrass loss, and/or be exacerbated by other stressors.
  - Herbivory
  - Invertebrates
  - Genetic Diversity Loss
  - Seagrass Competition
  - Pathogens
  - Microbial Dysbiosis

**References**

Amundrud, S. L., Srivastava, D. S., & O'Connor, M. I. (2015). Indirect effects of predators control herbivore richness and abundance in a benthic eelgrass (Zostera marina) mesograzer community. *Journal of Animal Ecology*, 84(4), 1092-1102.

Aoki, L. R., & McGlathery, K. J. (2018). Restoration enhances denitrification and DNRA in subsurface sediments of Zostera marina seagrass meadows. *Marine Ecology Progress Series,* 602, 87-102.

Beem, N. T., & Short, F. T. (2009). Subtidal eelgrass declines in the Great Bay estuary, New Hampshire and Maine, USA. *Estuaries and Coasts*, *32*, 202-205.

Borowitzka, M. A., Lavery, P. S., & Keulen, M. (2006). Epiphytes of seagrasses. *Seagrasses: Biology, ecology and conservation*, 441-461.

Brakel, J., Jakobsson-Thor, S., Bockelmann, A. C., & Reusch, T. B. (2019). Modulation of the eelgrass–Labyrinthula zosterae interaction under predicted ocean warming, salinity change and light limitation. *Frontiers in Marine Science*, 6, 268.

Carman, M. R., Colarusso, P. D., Neckles, H. A., Bologna, P., Caines, S., Davidson, J. D., ... & Wong, M. C. (2019). Biogeographical patterns of tunicates utilizing eelgrass as substrate in the western North Atlantic between 39° and 47° north latitude (New Jersey to Newfoundland). *Management of Biological Invasions*, *10*(4).

Carroll, J. M., Furman, B. T., Jackson, L. J., Hunter, E. A., & Peterson, B. J. (2019). Propagule risk in a marine foundation species: Seascape effects on Zostera marina seed predation. *Journal of Ecology*, *107*(4), 1982-1994.

Collier, C. J., Langlois, L., Ow, Y., Johansson, C., Giammusso, M., Adams, M. P., ... & Uthicke, S. (2018). Losing a winner: thermal stress and local pressures outweigh the positive effects of ocean acidification for tropical seagrasses. *New Phytologist*, *219*(3), 1005-1017.

Conover, J. T. (1958). Seasonal growth of benthic marine plants as related to an estuarine environment. *University of Michigan*.

Dayan, F. E. (2019). Current status and future prospects in herbicide discovery. *Plants*, *8*(9), 341.

De Casabianca, M. L., Laugier, T., & Marinho-Soriano, E. (1997). Seasonal changes of nutrients in water and sediment in a Mediterranean lagoon with shellfish farming activity (Thau Lagoon, France). *ICES Journal of Marine Science*, *54*(5), 905-916.

Egea, L. G., Jimenez-Ramos, R., Hernandez, I., Bouma, T. J., & Brun, F. G. (2018). Effects of ocean acidification and hydrodynamic conditions on carbon metabolism and dissolved organic carbon (DOC) fluxes in seagrass populations. *PLoS one*, *13*(2), e0192402.

Elso, M. Z., Manent, P., & Robaina, R. R. (2018). Zostera noltii in the Canary Islands: A Genetic Description for Conservation Purposes. *Journal of Coastal Research*, *34*(5), 1129-1135.

Ferguson, A. J., Gruber, R., Potts, J., Wright, A., Welsh, D. T., & Scanes, P. (2017). Oxygen and carbon metabolism of Zostera muelleri across a depth gradient–Implications for resilience and blue carbon. *Estuarine, Coastal and Shelf Science*, *187*, 216-230.

Hahn, D. R. (2003). Alteration of microbial community composition and changes in decomposition associated with an invasive intertidal macrophyte. *Marine Bioinvasions: Patterns, Processes and Perspectives*, 45-51.

Hansen, J. C., & Reidenbach, M. A. (2013). Seasonal growth and senescence of a Zostera marina seagrass meadow alters wave-dominated flow and sediment suspension within a coastal bay. *Estuaries and Coasts*, *36*, 1099-1114.

Harlin, M. M., & Thorne-Miller, B. (1981). Nutrient enrichment of seagrass beds in a Rhode Island coastal lagoon. *Marine Biology*, 65, 221-229.

Hirst, A. J., Longmore, A. R., Ball, D., Cook, P. L. M., & Jenkins, G. P. (2016). Linking nitrogen sources utilised by seagrass in a temperate marine embayment to patterns of seagrass change during drought. *Marine Ecology Progress Series*, 549, 79-88.

Hootsmans, M. J. M., & Vermaat, J. E. (1985). The effect of periphyton-grazing by three epifaunal species on the growth of Zostera marina L. under experimental conditions. *Aquatic Botany*, *22*(1), 83-88.

Hurtado-McCormick, V., Kahlke, T., Petrou, K., Jeffries, T., Ralph, P. J., & Seymour, J. R. (2019). Regional and microenvironmental scale characterization of the Zostera muelleri seagrass microbiome. *Frontiers in microbiology*, *10*, 1011.

Iacarella, J. C., Adamczyk, E., Bowen, D., Chalifour, L., Eger, A., Heath, W., ... & Baum, J. K. (2018). Anthropogenic disturbance homogenizes seagrass fish communities. *Global change biology*, *24*(5), 1904-1918.

Inaba, N., Trainer, V. L., Onishi, Y., Ishii, K. I., Wyllie-Echeverria, S., & Imai, I. (2017). Algicidal and growth-inhibiting bacteria associated with seagrass and macroalgae beds in Puget Sound, WA, USA. *Harmful algae*, 62, 136-147.

Jaschinski, S., & Sommer, U. (2010). Positive effects of mesograzers on epiphytes in an eelgrass system. *Marine Ecology Progress Series*, *401*, 77-85.

Jiang, Z., Liu, S., Zhang, J., Zhao, C., Wu, Y., Yu, S., ... & Kumar, M. (2017). Newly discovered seagrass beds and their potential for blue carbon in the coastal seas of Hainan Island, South China Sea. *Marine pollution bulletin*, *125*(1-2), 513-521.

Johnson, A. J., Shields, E. C., Kendrick, G. A., & Orth, R. J. (2021). Recovery dynamics of the seagrass Zostera marina following mass mortalities from two extreme climatic events. *Estuaries and Coasts*, 44, 535-544.

Kappel, C. V. (2005). Losing pieces of the puzzle: threats to marine, estuarine, and diadromous species. *Frontiers in Ecology and the Environment*, *3*(5), 275-282.

Kotta, J., Möller, T., Orav-Kotta, H., & Pärnoja, M. (2014). Realized niche width of a brackish water submerged aquatic vegetation under current environmental conditions and projected influences of climate change. *Marine environmental research*, 102, 88-101.

Lee, K. S., & Dunton, K. H. (2000). Diurnal changes in pore water sulfide concentrations in the seagrass Thalassia testudinum beds: the effects of seagrasses on sulfide dynamics. *Journal of experimental marine biology and ecology*, *255*(2), 201-214.

Lemley, D. A., Snow, G. C., & Human, L. R. D. (2014). The decomposition of estuarine macrophytes under different temperature regimes. *Water SA*, *40*(1), 117-124.

Lewis, L. S., & Anderson, T. W. (2012). Top‐down control of epifauna by fishes enhances seagrass production. *Ecology*, *93*(12), 2746-2757.

Lewis, J. T., & Boyer, K. E. (2014). Grazer functional roles, induced defenses, and indirect interactions: implications for eelgrass restoration in San Francisco Bay. *Diversity*, *6*(4), 751-770.

Liu, W., Pearce, C. M., & Dovey, G. (2015). Assessing potential benthic impacts of harvesting the Pacific geoduck clam Panopea generosa in intertidal and subtidal sites in British Columbia, Canada. *Journal of Shellfish Research*, *34*(3), 757-775.

McGlathery, K. J., Reidenbach, M. A., D'odorico, P. A. O. L. O., Fagherazzi, S., Pace, M. L., & Porter, J. H. (2013). Nonlinear dynamics and alternative stable states in shallow coastal systems. *Oceanography*, 26(3), 220-231.

McKone, K. L., & Tanner, C. E. (2009). Role of salinity in the susceptibility of eelgrass Zostera marina to the wasting disease pathogen Labyrinthula zosterae. *Marine Ecology Progress Series*, 377, 123-130.

McMahon, K., Nash, S. B., Eaglesham, G., Müller, J. F., Duke, N. C., & Winderlich, S. (2005). Herbicide contamination and the potential impact to seagrass meadows in Hervey Bay, Queensland, Australia. *Marine Pollution Bulletin*, *51*(1-4), 325-334.

Miller, C. A., Yang, S., & Love, B. A. (2017). Moderate increase in TCO2 enhances photosynthesis of seagrass Zostera japonica, but not Zostera marina: Implications for acidification mitigation. *Frontiers in Marine Science*, *4*, 228.

Momota, K., & Nakaoka, M. (2017). Influence of different types of sessile epibionts on the community structure of mobile invertebrates in an eelgrass bed. *PeerJ*, 5, e2952.

Mylona, Z., Panteris, E., Kevrekidis, T., & Malea, P. (2020). Silver nanoparticle toxicity effect on the seagrass Halophila stipulacea. *Ecotoxicology and Environmental Safety*, *189*, 109925.

Palacios, S. L., & Zimmerman, R. C. (2007). Response of eelgrass Zostera marina to CO2 enrichment: possible impacts of climate change and potential for remediation of coastal habitats. *Marine Ecology Progress Series*, *344*, 1-13.

Paulo, D., Diekmann, O., Ramos, A. A., Alberto, F., & Serrão, E. A. (2019). Sexual reproduction vs. clonal propagation in the recovery of a seagrass meadow after an extreme weather event. *Sci. Mar*, *83*, 357-363.

Pazzaglia, J., Reusch, T. B., Terlizzi, A., Marín‐Guirao, L., & Procaccini, G. (2021). Phenotypic plasticity under rapid global changes: The intrinsic force for future seagrasses survival. *Evolutionary Applications*, *14*(5), 1181-1201.

Pedersen, O., Binzer, T., & Borum, J. (2004). Sulphide intrusion in eelgrass (Zostera marina L.). *Plant, Cell & Environment*, *27*(5), 595-602.

Pellikaan, G. C., & Nienhuis, P. H. (1988). Nutrient uptake and release during growth and decomposition of eelgrass, Zostera marina L., and its effects on the nutrient dynamics of Lake Grevelingen. *Aquatic botany*, 30(3), 189-214.

Procaccini, G., Olsen, J. L., & Reusch, T. B. (2007). Contribution of genetics and genomics to seagrass biology and conservation. *Journal of Experimental Marine Biology and Ecology*, *350*(1-2), 234-259.

Rose, C. D., & Dawes, C. J. (1999). Effects of community structure on the seagrass Thalassia testudinum. *Marine Ecology Progress Series*, 184, 83-95.

Ruesink, J. L., Feist, B. E., Harvey, C. J., Hong, J. S., Trimble, A. C., & Wisehart, L. M. (2006). Changes in productivity associated with four introduced species: ecosystem transformation of a ‘pristine’estuary. *Marine Ecology Progress Series*, *311*, 203-215.

Ruesink, J. L., Hong, J. S., Wisehart, L., Hacker, S. D., Dumbauld, B. R., Hessing-Lewis, M., & Trimble, A. C. (2010). Congener comparison of native (Zostera marina) and introduced (Z. japonica) eelgrass at multiple scales within a Pacific Northwest estuary. *Biological Invasions*, *12*, 1773-1789.

Sand-Jensen, K. (1975). Biomass, net production and growth dynamics in an eelgrass (Zostera marina L.) population in Vellerup Vig, Denmark. *Ophelia*, 14(1-2), 185-201

Seymour, J. R., Laverock, B., Nielsen, D. A., Trevathan-Tackett, S. M., & Macreadie, P. I. (2018). The microbiology of seagrasses. In *Seagrasses of Australia: Structure, Ecology and Conservation* (pp. 343-392). Cham: Springer International Publishing.

Sola, I., Zarzo, D., Carratalá, A., Fernández-Torquemada, Y., de-la-Ossa-Carretero, J. A., Del-Pilar-Ruso, Y., & Sánchez-Lizaso, J. L. (2020). Review of the management of brine discharges in Spain. *Ocean & Coastal Management*, 196, 105301.

Sugimoto, K., Nakano, Y., Okuda, T., Nakai, S., Nishijima, W., & Okada, M. (2017). Coexistence between Zostera marina and Zostera japonica in seagrass beds of the Seto Inland Sea, Japan. *Journal of Ecology and Environment*, *41*, 1-9.

Terrados, J., Duarte, C. M., Kamp-Nielsen, L., Agawin, N. S. R., Gacia, E., Lacap, D., ... & Greve, T. (1999). Are seagrass growth and survival constrained by the reducing conditions of the sediment?. *Aquatic Botany*, 65(1-4), 175-197.

Turschwell, M. P., Connolly, R. M., Dunic, J. C., Sievers, M., Buelow, C. A., Pearson, R. M., ... & Brown, C. J. (2021). Anthropogenic pressures and life history predict trajectories of seagrass meadow extent at a global scale. *Proceedings of the National Academy of Sciences*, *118*(45), e2110802118.

van Lent, F., Verschuure, J. M., & van Veghel, M. L. (1995). Comparative study on populations of Zostera marina L.(eelgrass): in situ nitrogen enrichment and light manipulation. *Journal of Experimental Marine Biology and Ecology*, 185(1), 55-76.

Verhagen, J. H. G., & Nienhuis, P. H. (1983). A simulation model of production, seasonal changes in biomass and distribution of eelgrass (Zostera marina) in Lake Grevelingen. *Marine Ecology Progress Series*, 10(2), 187-195.

Williams, S. L. (2001). Reduced genetic diversity in eelgrass transplantations affects both population growth and individual fitness. *Ecological Applications*, *11*(5), 1472-1488.

Zimmerman, R. C., Smith, R. D., & Alberte, R. S. (1987). Is growth of eelgrass nitrogen limited? A numerical simulation of the effects of light and nitrogen on the growth dynamics of Zostera marina. *Marine Ecology Progress Series,* 41(2).
