# Supplementary material for: Understanding trends in Zostera research, stressors, and response variables: a global systematic review of the seagrass genus
Source: PeerJ. 2025 Apr 17;13:e19209. doi: 10.7717/peerj.19209 (PMC12009562; doi:10.7717/peerj.19209)
Supplement: Supplemental Information 10 [file peerj-13-19209-s010.docx]

# **Supplemental Information 6. Descriptions and examples of response variables measured in full text articles studying seagrasses.**

**SI 6.1 Plant Response Variables**

- Shoot Density
  - A single measurement in time of plant length, biomass, leaf size (Lee et al., 2007).
- Growth
  - A measured change over time in plant length, biomass, or new leaves (Hughes et al., 2013).
- Physiological Measures
  - Measured plant contents of Chlorophyll A, Carbohydrates, Nutrients (Rodrigues & Pardal, 2015).
- Reproductive Output
  - A measurement of a plant’s seeds, flowering, or clonal growth (Carroll et al., 2019).
- Photosynthetic Output
  - A measurement of a plant’s photosynthetic performance, pigments, or photosynthesis process (Kumar et al., 2017).

**SI 6.2 Community Response Variables**

- Invertebrates
  - Measurements of invertebrate responses, diversity, or functionality within seagrass meadows or in relation to seagrass meadows (Niquil et al., 2014).
- Microbial Diversity
  - Measurements of the microbial community within or directly surrounding seagrass plants (Twigg et al., 2020).
- Distribution
  - Measurements of meadow surface area cover, models of current and past ranges, measurements of meadow gradients (Yang, HilleRisLambers, & Ruesink, 2016).
- Epiphytes
  - Measurements of epiphyte density, diversity, presence within seagrass meadows and plants (Prado, 2018).
- Organismal Diversity
  - Measurements of variation and abundance of species within or related to seagrass communities (Do et al., 2011 and Kennish, Haag, & Sakowicz, 2008).
- Macroalgae
  - Measurements of macroalgae presence or influence on seagrass communities. Measurement types included cover, presence/absence, density. Examples of macroalgae types included *Spyridia filamentosa*, *Gracilaria tikvahiae*, *Champia parvula*, and *Ulva lactuca* (Kennish, Haag, & Sakowicz, 2008).
- Seagrass competition
  - Seagrass competition within the same species/genus or between species of seagrass. Examples include *Zostera marina*’s competition with the invasive *Zostera japonica* (Bando, 2006).
- Chlorophyll A
  - Proxy measurement of phytoplankton presence, productivity in a seagrass community (Best & Stachowicz, 2012).

**SI 6.3 Environmental Response Variables**

- Sediment Characteristics
  - Measurements of CaCO3, Grain Size, Carbon Redox Potential, and Carbon Storage as related to sediment (Spivak, et al., 2007, Suykerbuyk et al., 2016 and Serrano et al., 2020).
- Water Column Nutrients
  - Measurements of nutrients (dissolved organic nitrogen, total nitrogen, chlorophyll a) within the water column (Moksnes et al., 2018 and Tomasko & Lapointe, 1991).
- Sediment Nutrients
  - Measurements of nutrient parameters (sulfide, carbon storage, total nitrogen, phosphorus) within the sediment (McGlathery, 2001 and Sfriso & Marcomini, 1999).
- Water Column Salinity
  - Measurements of the amount of dissolved salts in the water column, typically used as a water quality parameter, or measurement of water flow (Lillebø et al., 2005 and Howarth et al., 2014).
- Water Column Temperature
  - Measurement of water column temperature, viewed as a parameter of water quality. Often measured with irradiance (Nakayama et al., 2020 and Jarvis, Brush, & Moore, 2014).
- Water Column Dissolved Oxygen
  - Measurements of variation in dissolved oxygen. Often used as a water quality indicator, measurement of metabolism or a proxy for carbon cycling within an ecosystem (Rheuban, Berg, & McGlathery, 2014 and Gustafsson & Norkko, 2016).
- Water Column pH
  - Measurements of acidity or alkalinity in the water column, a common water quality parameter. Water column pH can also be used as a proxy for metabolism or photosynthetic activity (Qu et al., 2006 and Burkholder, Mason, & Glasgow, 1992).
- Water Column Turbidity
  - Measurements of visibility or light penetration in the water column. Typically used to look at hydrodynamics, sediment in the water column, or alterations of light due to macroalgae presence (Gustafsson & Boström, 2014 and Lillebø et al., 2007).

**References**

Bando, K. J. (2006). The roles of competition and disturbance in a marine invasion. *Biological Invasions*, 8, 755-763.

Best, R. J., & Stachowicz, J. J. (2012). Trophic cascades in seagrass meadows depend on mesograzer variation in feeding rates, predation susceptibility, and abundance. *Marine Ecology Progress Series*, 456, 29-42.

Burkholder, J. M., Mason, K. M., & Glasgow Jr, H. B. (1992). Water-column nitrate enrichment promotes decline of eelgrass Zostera marina: evidence from seasonal mesocosm experiments. *Marine ecology progress series. Oldendorf*, *81*(2), 163-178.

Carroll, J. M., Furman, B. T., Jackson, L. J., Hunter, E. A., & Peterson, B. J. (2019). Propagule risk in a marine foundation species: Seascape effects on Zostera marina seed predation. *Journal of Ecology*, 107(4), 1982-1994.

Do, V. T., de Montaudouin, X., Lavesque, N., Blanchet, H., & Guyard, H. (2011). Seagrass colonization: Knock-on effects on zoobenthic community, populations and individual health. *Estuarine, Coastal and Shelf Science*, 95(4), 458-469.

Gustafsson, C., & Boström, C. (2014). Algal mats reduce eelgrass (Zostera marina L.) growth in mixed and monospecific meadows. *Journal of experimental marine biology and ecology*, *461*, 85-92.

Gustafsson, C., & Norkko, A. (2016). Not all plants are the same: Exploring metabolism and nitrogen fluxes in a benthic community composed of different aquatic plant species. *Limnology and Oceanography*, *61*(5), 1787-1799.

Howarth, R. W., Hayn, M., Marino, R. M., Ganju, N., Foreman, K., McGlathery, K., ... & Walker, J. D. (2014). Metabolism of a nitrogen-enriched coastal marine lagoon during the summertime. *Biogeochemistry*, *118*, 1-20.

Hughes, B. B., Eby, R., Van Dyke, E., Tinker, M. T., Marks, C. I., Johnson, K. S., & Wasson, K. (2013). Recovery of a top predator mediates negative eutrophic effects on seagrass. *Proceedings of the National Academy of Sciences*, 110(38), 15313-15318.

Jarvis, J. C., Brush, M. J., & Moore, K. A. (2014). Modeling loss and recovery of Zostera marina beds in the Chesapeake Bay: the role of seedlings and seed-bank viability. *Aquatic Botany*, *113*, 32-45.

Kennish, M. J., Haag, S. M., & Sakowicz, G. P. (2008). Seagrass demographic and spatial habitat characterization in Little Egg Harbor, New Jersey, using fixed transects. *Journal of Coastal Research*, (10055), 148-170.

Kumar, M., Padula, M. P., Davey, P., Pernice, M., Jiang, Z., Sablok, G., ... & Ralph, P. J. (2017). Proteome analysis reveals extensive light stress-response reprogramming in the seagrass Zostera muelleri (Alismatales, Zosteraceae) metabolism. *Frontiers in Plant Science*, 7, 2023.

Lee, K. S., Park, J. I., Kim, Y. K., Park, S. R., & Kim, J. H. (2007). Recolonization of Zostera marina following destruction caused by a red tide algal bloom: the role of new shoot recruitment from seed banks. *Marine Ecology Progress Series*, 342, 105-115.

Lillebø, A. I., Neto, J. M., Martins, I., Verdelhos, T., Leston, S., Cardoso, P. G., ... & Pardal, M. A. (2005). Management of a shallow temperate estuary to control eutrophication: the effect of hydrodynamics on the system’s nutrient loading. *Estuarine, coastal and shelf science*, 65(4), 697-707.

Lillebø, A. I., Teixeira, H., Pardal, M. A., & Marques, J. C. (2007). Applying quality status criteria to a temperate estuary before and after the mitigation measures to reduce eutrophication symptoms. *Estuarine, Coastal and Shelf Science*, *72*(1-2), 177-187.

McGlathery, K. J. (2001). Macroalgal blooms contribute to the decline of seagrass in nutrient‐enriched coastal waters. *Journal of Phycology*, 37(4), 453-456.

Moksnes, P. O., Eriander, L., Infantes, E., & Holmer, M. (2018). Local regime shifts prevent natural recovery and restoration of lost eelgrass beds along the Swedish west coast. *Estuaries and coasts*, 41, 1712-1731.

Nakayama, K., Komai, K., Tada, K., Lin, H. C., Yajima, H., Yano, S., ... & Tsai, J. W. (2020). Modeling dissolved inorganic carbon considering submerged aquatic vegetation. *Ecological Modelling*, *431*, 109188.

Niquil, N., Baeta, A., Marques, J. C., Chaalali, A., Lobry, J., & Patrício, J. (2014). Reaction of an estuarine food web to disturbance: Lindeman’s perspective. *Marine Ecology Progress Series,* 512, 141-154.

Prado, P. (2018). Seagrass epiphytic assemblages are strong indicators of agricultural discharge but weak indicators of host features. *Estuarine, Coastal and Shelf Science*, 204, 140-148.

Qu, W., Morrison, R. J., West, R. J., & Su, C. (2006). Organic matter and benthic metabolism in Lake Illawarra, Australia. *Continental shelf research*, *26*(15), 1756-1774.

Rheuban, J. E., Berg, P., & McGlathery, K. J. (2014). Multiple timescale processes drive ecosystem metabolism in eelgrass (Zostera marina) meadows. *Marine Ecology Progress Series*, *507*, 1-13.

Rodrigues, E. T., & Pardal, M. Â. (2015). Primary productivity temporal fluctuations in a nutrient-rich estuary due to climate-driven events. *Estuaries and coasts*, 38, 1-12.

Serrano, O., Rozaimi, M., Lavery, P. S., & Smernik, R. J. (2020). Organic chemistry insights for the exceptional soil carbon storage of the seagrass Posidonia australis. *Estuarine, Coastal and Shelf Science*, 237, 106662.

Sfriso, A., & Marcomini, A. (1999). Macrophyte production in a shallow coastal lagoon. Part II: Coupling with sediment, SPM and tissue carbon, nitrogen and phosphorus concentrations. *Marine Environmental Research*, 47(3), 285-309.

Spivak, A. C., Canuel, E. A., Duffy, J. E., & Richardson, J. P. (2007). Top‐down and bottom‐up controls on sediment organic matter composition in an experimental seagrass ecosystem. *Limnology and Oceanography*, 52(6), 2595-2607.

Suykerbuyk, W., Bouma, T. J., Govers, L. L., Giesen, K., de Jong, D. J., Herman, P., ... & van Katwijk, M. M. (2016). Surviving in changing seascapes: sediment dynamics as bottleneck for long-term seagrass presence. *Ecosystems*, 19, 296-310

Tomasko, D. A., & Lapointe, B. E. (1991). Productivity and biomass of Thalassia testudinum as related to water column nutrient availability and epiphyte levels: field observations and experimental studies. *Marine ecology progress series*, 9-17.

Twigg, I. M., Baltar, F., Hall, J. R., & Hepburn, C. D. (2020). Revealing hydrogen peroxide as an external stressor in macrophyte-dominated coastal ecosystems. *Oecologia*, 193, 583-591.

Yang, S., HilleRisLambers, J., & Ruesink, J. L. (2016). Reversal of intraspecific interactions by an ecosystem engineer leads to variable seedling success along a stress gradient. *Marine Ecology Progress Series*, 543, 163-171.
