# Supplementary figures and images for: Understanding trends in Zostera research, stressors, and response variables: a global systematic review of the seagrass genus
Source: PeerJ. 2025 Apr 17;13:e19209. doi: 10.7717/peerj.19209 (PMC12009562; doi:10.7717/peerj.19209)

# Abstract Screening Decision Tree

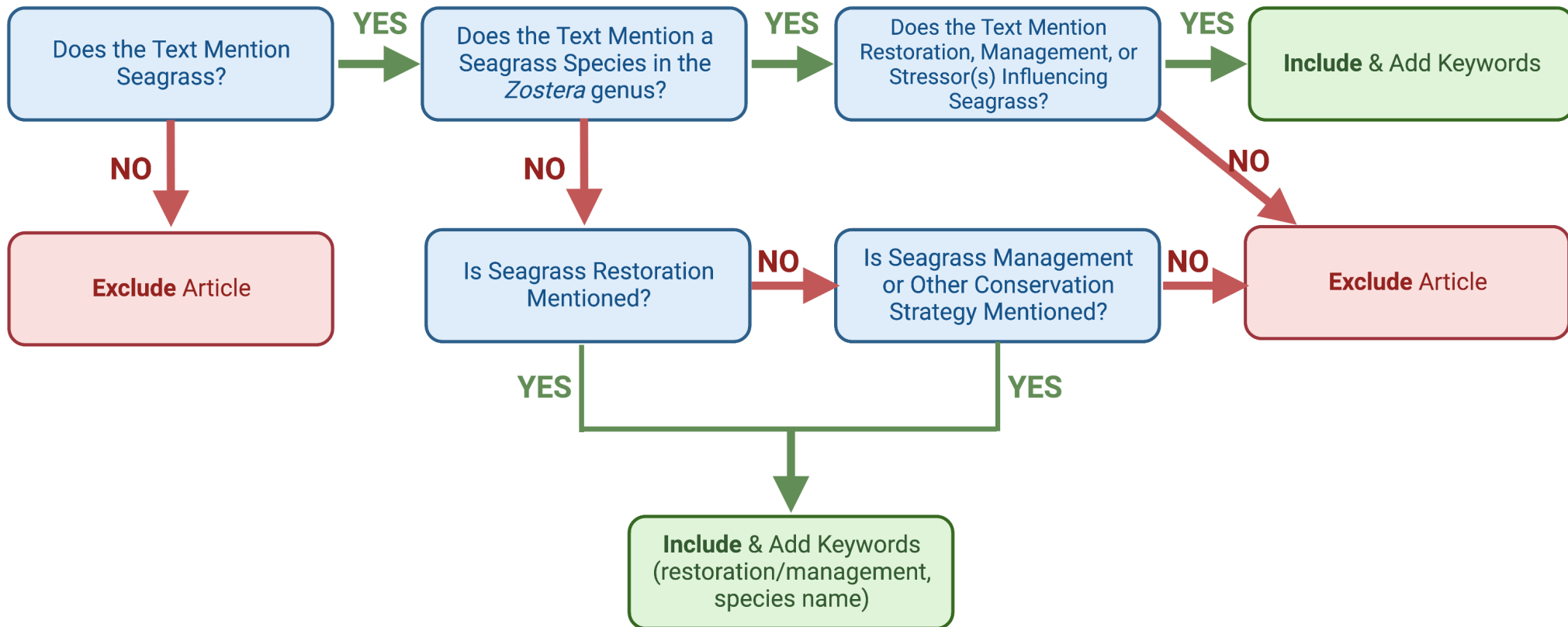

Supplement: Supplemental Information 13 — All article screeners were trained using this workflow, inclusion or exclusion of each article required two screeners to use this tree and arrive at the same conclusion for exclusion or inclusion of an article. [file peerj-13-19209-s013.pdf]

# Full Text Screening Decision Tree

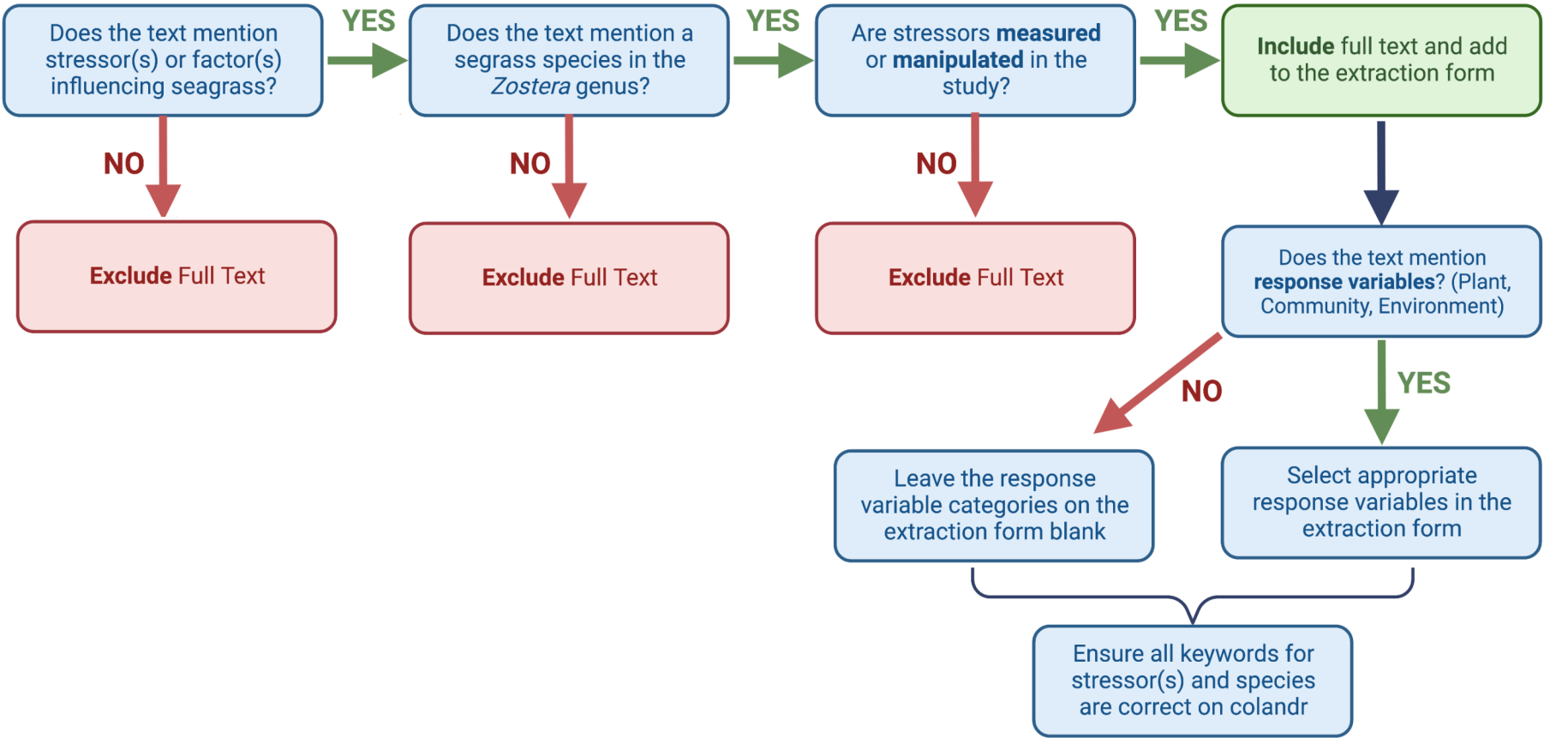

Supplement: Supplemental Information 14 — All article screeners were trained using this workflow in addition to 10 practice articles; inclusion or exclusion of each article required a screener to use this tree, make a decision to include or exclude the article, then extract data from the article if an include decision was made. [file peerj-13-19209-s014.pdf]

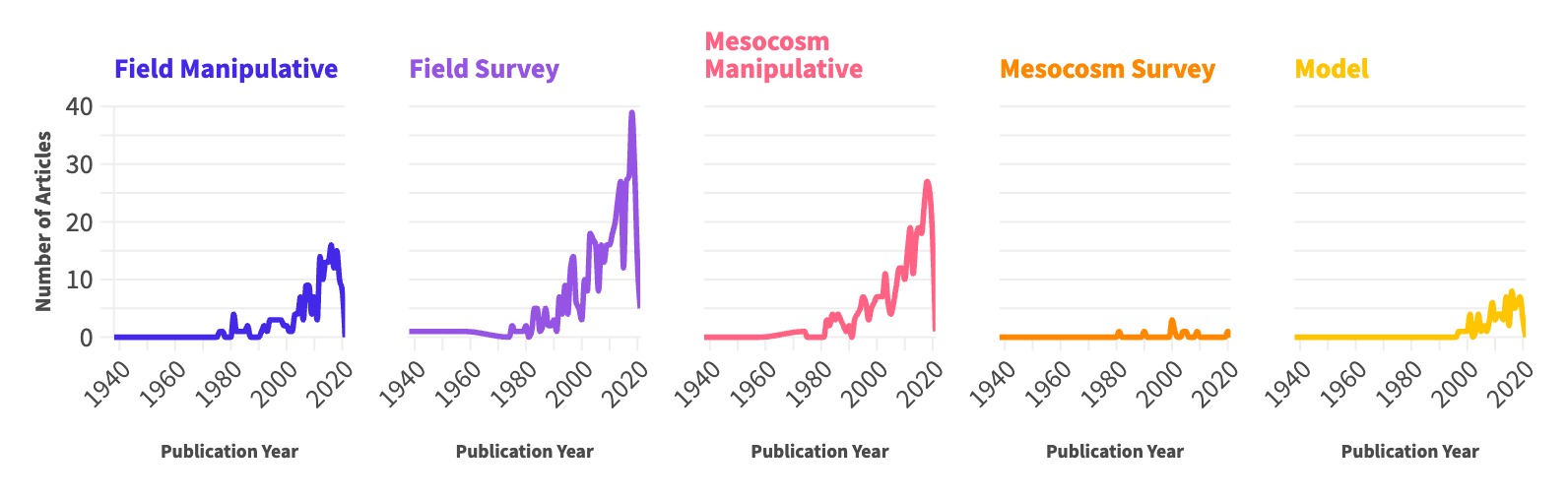

Supplement: Supplemental Information 16 — Each of the five study type and design combinations are displayed with data points for the publication years 1938–2021. Most 2021 publications were pre-prints at the time of literature search, and article numbers are only representative up until the date of the search execution, meaning the 2020 and 2021 numbers may not be completely represented by our review. [file peerj-13-19209-s016.jpeg]

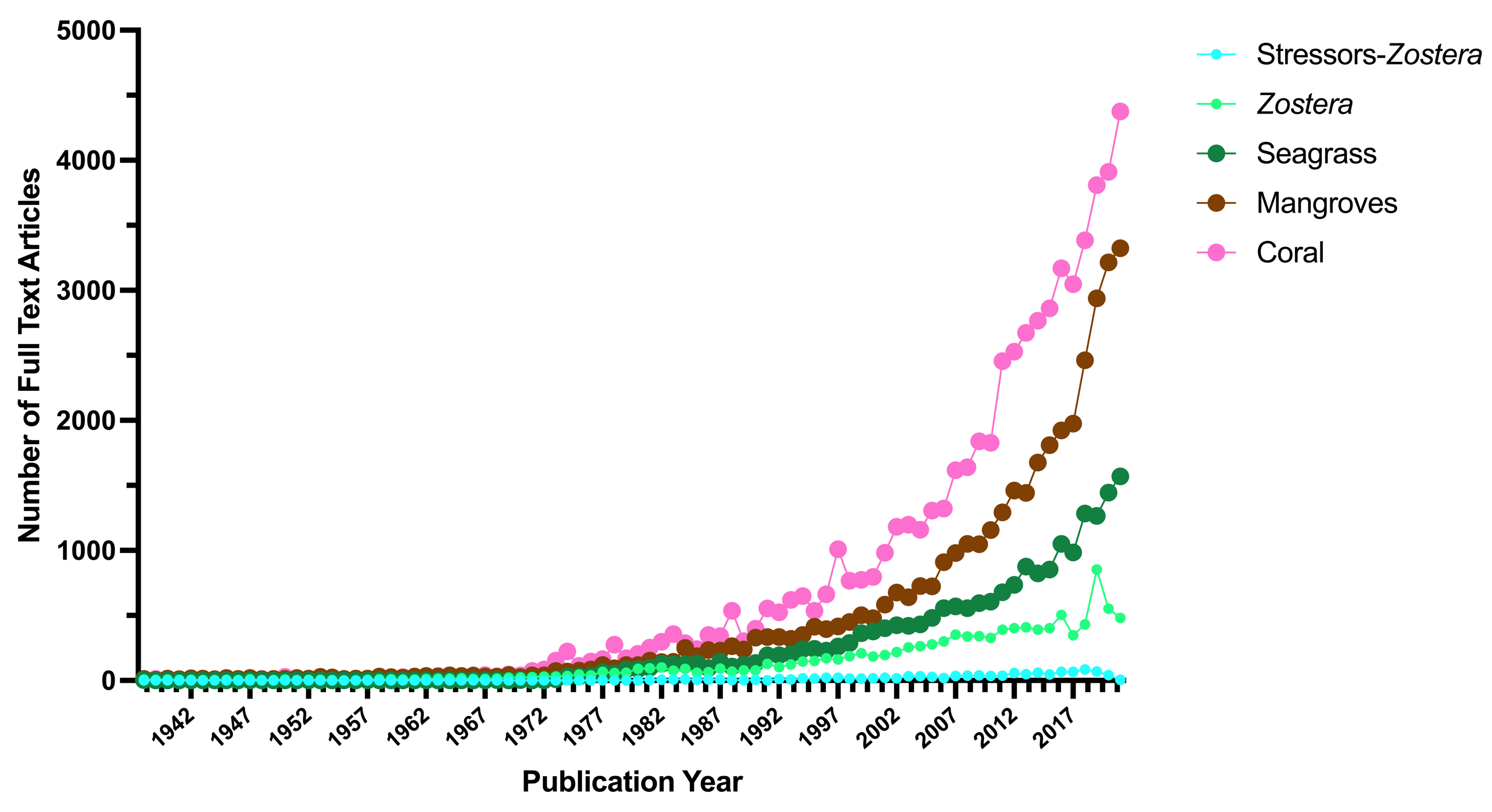

Supplement: Supplemental Information 17 — Number of articles per publication year for included full-text articles (Zostera-stressor), and a Web of Science search for: genus (Zostera), seagrass overall (Seagrass), and two other coastal ecosystems (Coral, Mangroves). Article numbers per publication year were obtained from a Web of Science search for each respective term. Most 2021 publications were pre-prints at the time of literature search, and article numbers are only representative up until the date of the search execution, meaning the 2020 and 2021 numbers may not be completely represented by our review. [file peerj-13-19209-s017.jpg]

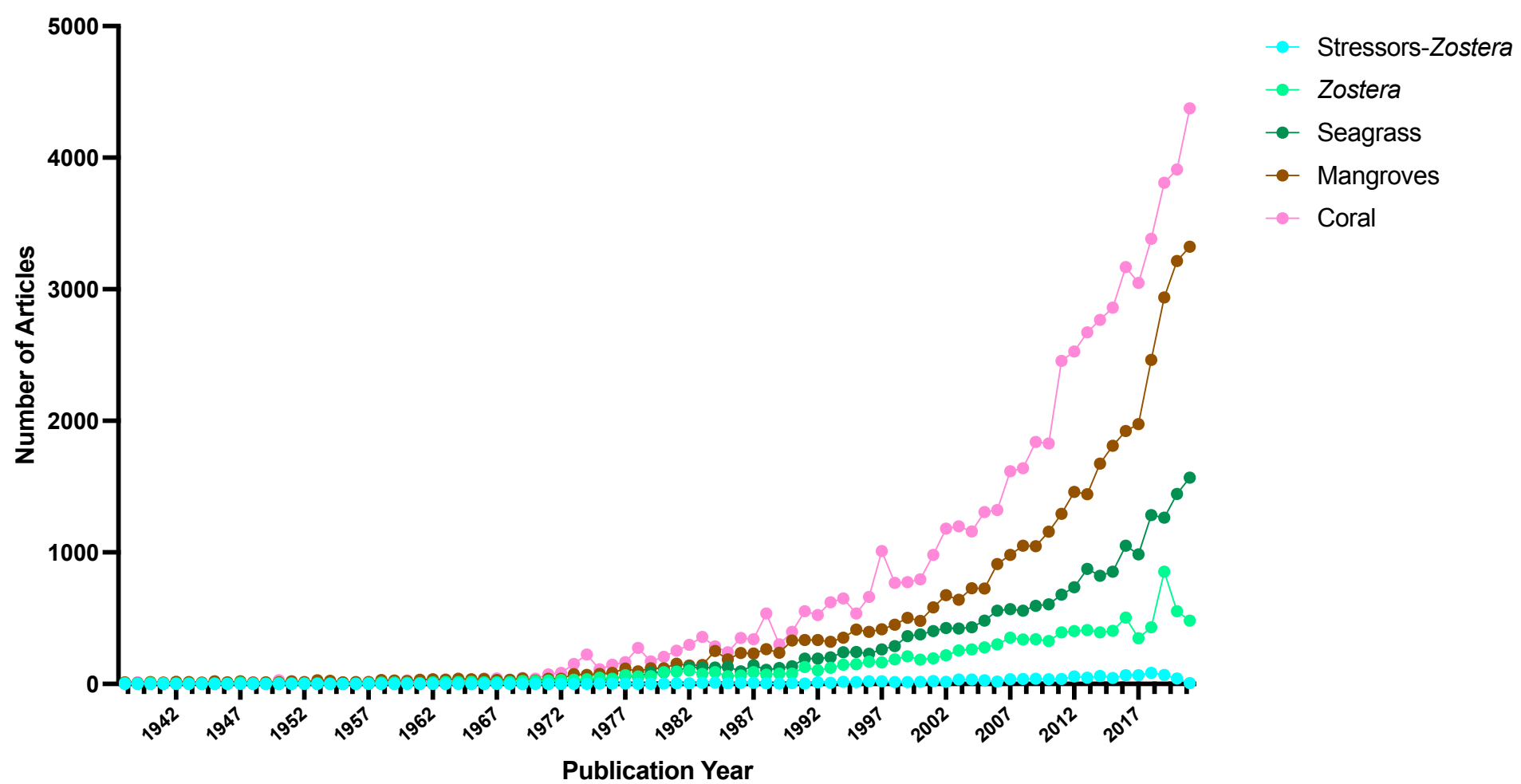

Supplement: Supplemental Information 18 — (A) Publication years for articles that measured one stressor, compared to articles that measured more than one stressor. (B) Publication years for articles broken down by the exact number of stressors recorded (1, 2, 3, 4+). Most 2021 publications were pre-prints at the time of literature search, and article numbers are only representative up until the date of the search execution, meaning the 2020 and 2021 numbers may not be completely represented by our review. [file peerj-13-19209-s018.pdf]

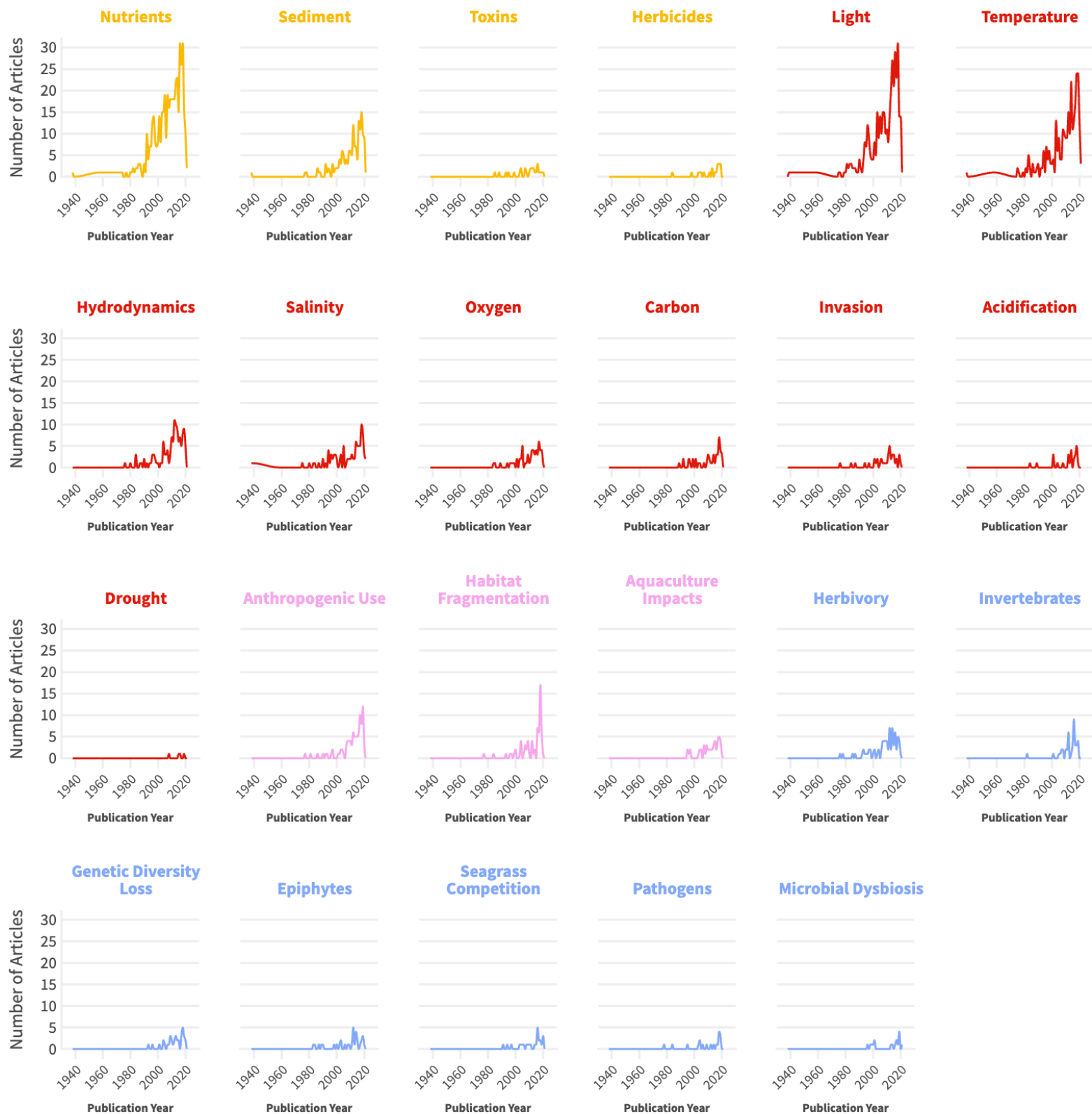

Supplement: Supplemental Information 19 — Grid ordered by total articles for each individual stressor within the four umbrella categories, highest to lowest. Stressors colored by umbrella categories: pollution (orange), climate change (red), increased anthropogenic presence (pink), and intrinsic factors (blue). [file peerj-13-19209-s019.pdf]

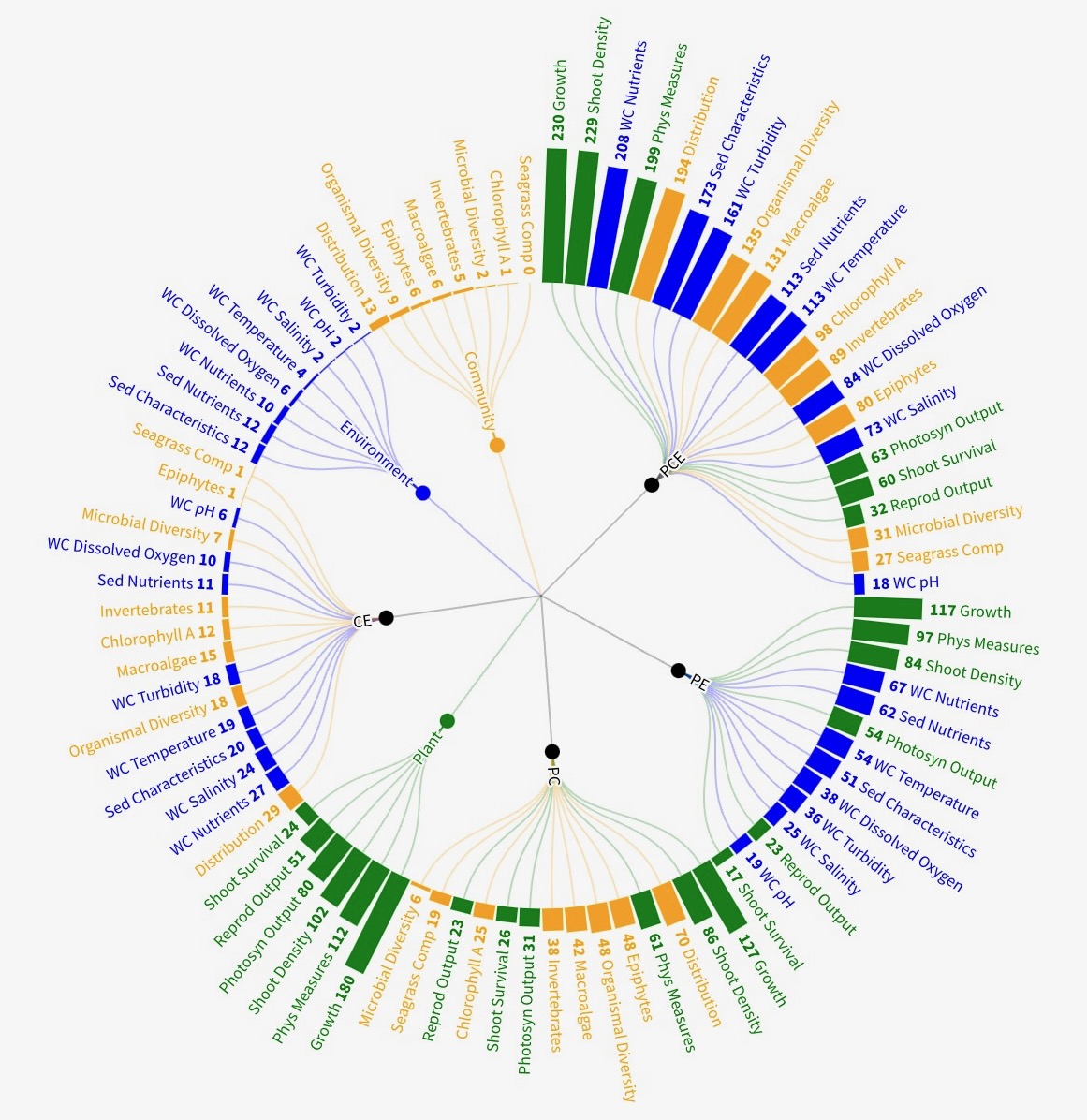

Supplement: Supplemental Information 20 — Individual type and counts of each response variable were sorted into categories of plant, community, and environment recorded in combination by each included article. Combinations included: Plant, Community, or Environment only; Plant & Community (PC); Plant & Environment (PE); Community & Environment (CE); or Plant, Community, & Environment (PCE). [file peerj-13-19209-s020.jpeg]
