# Supplementary material for: Understanding trends in Zostera research, stressors, and response variables: a global systematic review of the seagrass genus
Source: PeerJ. 2025 Apr 17;13:e19209. doi: 10.7717/peerj.19209 (PMC12009562; doi:10.7717/peerj.19209)
Supplement: Supplemental Information 15 — All extraction fields for data extraction during full-text article review. Fields were contained in the form of an excel sheet, where reviewers had drop-down options for selection, eliminating variation between reviewers. Supplemental information S4 also contains an example of the data extraction sheet. [file peerj-13-19209-s015.pdf]

Full-Text Data Extraction Fields

Citation Information

- Title
- Authors
- Year
- Date Screened
- Name of Individual Screening
- Article

Study Details

- Study Design
  - Field
  - Mesocosm
  - Model
- Study Type
- Survey
- Manipulative
- *Zostera* species
  - angustifolia
  - asiatica
  - caespitosa
  - capensis
  - capricornii
  - caulescens
  - chilensis
  - japonica
  - marina
  - mucronata
  - muelleri
  - nigricaulis
  - noltii
  - tasmanica
- Location Name
- Location (GPS)
- Latitude
- Longitude

Study Details

- Acidification
- Anthropogenic Use
- Aquaculture Impacts
- Carbon
- Drought
- Epiphytes
- Genetic Diversity Loss
- Habitat Fragmentation
- Herbicides
- Herbivory
- Hydrodynamics
- Invasion
- Invertebrates
- Light
- Microbial Dysbiosis
- Nutrients
- Oxygen
- Pathogens
- Salinity
- Seagrass competition
- Sediment
- Temperature
- Toxins

Plant Response Variables

- Growth
- Photosynthetic Output
- Physiological Measures
- Reproductive Output
- Shoot Density

Community Response Variables

- Chlorophyll A
- Distribution
- Epiphytes
- Invertebrates
- Macroalgae
- Microbial Diversity
- Organismal Diversity
- Seagrass competition

Environmental Response Variables

- Sediment Characteristics Sediment Nutrients
- Water Column Dissolved Oxygen
- Water Column Nutrients
- Water Column pH
- Water Column Salinity
- Water Column Temperature
- Water Column Turbidity

Additional Fields

- Growth
- Photosynthetic Output
- Physiological Measures
- Reproductive Output
- Shoot Density
